# Iron(II) Spin Crossover Coordination Polymers Derived From a Redox Active Equatorial Tetrathiafulvalene Schiff-Base Ligand

**DOI:** 10.3389/fchem.2021.692939

**Published:** 2021-08-02

**Authors:** Ya-Ru Qiu, Long Cui, Jing-Yuan Ge, Mohamedally Kurmoo, Guijun Ma, Jian Su

**Affiliations:** ^1^ School of Physical Science and Technology, ShanghaiTech University, Shanghai, China; ^2^ State Key Laboratory of Coordination Chemistry, School of Chemistry and Chemical Engineering, Nanjing National Laboratory of Microstructures, Nanjing University, Nanjing, China; ^3^ College of Chemistry and Materials Engineering, Wenzhou University, Wenzhou, China; ^4^ Institut de Chimie de Strasbourg, CNRS-UMR 7177 Université de Strasbourg, Strasbourg, France

**Keywords:** coordination polymers, tetrathiafulvalene, schiff-base ligand, spin-crossover, fluorescence

## Abstract

Two polymorphic Fe^II^ coordination polymers [Fe^II^L (TPPE)_0.5_] **1**) and [(Fe^II^_3_L_3_ (TPPE)_1.5_)] **2**), were obtained from a redox-active tetrathiafulvalene (TTF) functionalized ligand [H_2_L = 2,2’-(((2-(4,5-bis-(methylthio)-1,3-dithiol-2-ylidene)benzo(d) (1,3) dithiole-5,6-diyl)bis-(azanediyl))bis-(meth anylylidene)) (2E,2E')-bis(3-oxobutanoate)] and a highly luminescent connector {TPPE = 1,1,2,2-tetrakis[4-(pyridine-4-yl)phenyl]-ethene}. Complex **1** has a layered structure where the TPPE uses its four diverging pyridines from the TPPE ligand are coordinated by the *trans* positions to the flat TTF Schiff-base ligand, and complex **2** has an unprecedented catenation of layers within two interpenetrated frameworks. These coordination polymers reserved the redox activity of the TTF unit. Complex **1** shows gradual spin transition behavior without hysteresis. And the fluorescence intensity of TPPE in **1** changes in tandem with the spin crossover (SCO) transition indicating a possible interplay between fluorescence and SCO behavior.

## Introduction

Among numerous multifunctional materials, spin crossover (SCO) complexes exhibiting switching between low-spin (LS) and high-spin (HS) states, are one of the most fashionable examples of molecular bistability (Smith et al., 2003; Weber, 2009; Tao et al., 2012; Harding et al., 2016; Ni et al., 2017). The SCO behavior can be effected by different external incentives such as temperature, pressure, or light radiation (Hoshino et al., 2012; Zheng et al., 2018). The change in the spin-state brings about the attractive shift in structural, optical, and electrical properties making SCO systems absorbing for applications in physics, chemistry and materials science (Gütlich et al., 2000; Halcrow, 2011; Kepp, 2016). Recently, SCO systems displaying multifunctionality [such as electrical conductivity (Wang et al., 2018a; De la Barrera et al., 2018) or optical behaviors (Delgado et al., 2018; Lochenie et al., 2018)] have been focused, while the challenges still persist in developing fluorescent SCO complexes.

In fact, it is a wise choice to employ coordination connectors with distinct configuration and performance in order to incorporate spin transition and fluorescence. The wonderful 1,1,2,2-tetrakis[4-(pyridine-4-yl)phenyl]-ethene (TPPE) ligand, which includes four pyridine rings around a central ethylene with a diverting “propeller” configuration, acts as a bridging ligand to form polymeric networks (Kapadia et al., 2011a; Huang et al., 2012; Icli et al., 2012; Pigge et al., 2013). Additionally, TPPE and its derivatives have extended π-conjugation which may give rise to aggregation-induced emission (AIE); consequently, an increasing number of corresponding reports has emerged (Kapadia et al., 2011b; Gong et al., 2014). Recently, our laboratory constructed a two-dimensional (2D) Fe^II^ coordination polymer {[Fe(L)](TPPE)_0.5_·3CH_3_OH}_n_ (L is a N_2_O_2_^2−^ coordinating Schiff-base) showing the hysteretic SCO behavior of 25 K width, of which the correlation of SCO behavior and fluorescent properties were achieved (Ge et al., 2019).

**SCHEME 1:**
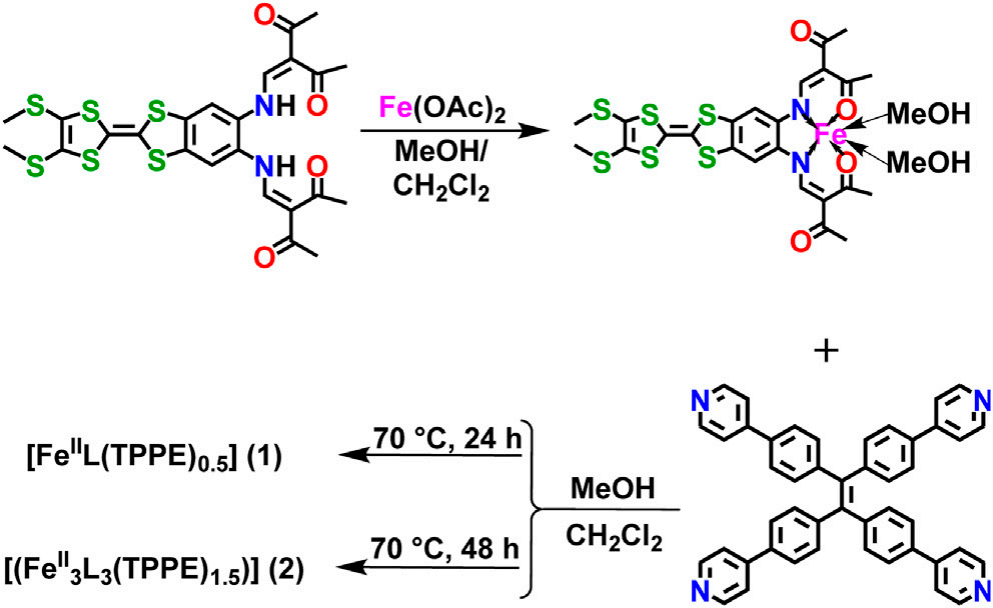
The synthetic method for TTF Schiff-base ligand (H_2_L), precursor [Fe^Ⅱ^L (CH_3_OH)_2_], [Fe^II^L (TPPE)_0.5_] (**1**) and [(Fe^II^_3_L_3_ (TPPE)_1.5_)] (**2**).

Besides to the luminescent connector, it is anoter excellent strategy to introduce functionalized Schiff-base ligand into the SCO and emission properties. Tetrathiafulvalene (TTF) is a sulphur rich, planar organic model with fourteen highly delocalized π electrons, which has a canonical redox-active core. TTF and its derivatives are readily functionalized to coordinate to a diverse range of magnetic centres and have been widely explored as a means of incorporating redox activity into a material (Su et al., 2017; Wang et al., 2017a; Schönfeld et al., 2020; Zappe et al., 2020). Up to now, a number of redox-active materials based on TTF have been studied (Su et al., 2017; Wang et al., 2017a; Wang et al., 2017b; Qiu et al., 2020; Schönfeld et al., 2021). Inspired by these results, we sought to introduce the luminescent ligand, TPPE, into Fe^II^ coordination polymers based on a TTF Schiff-base ligand (H_2_L = 2,2'-(((2-(4,5-bis (methylthio)-1,3-dithiol-2-ylidene)-benzo[*d*][1,3]di-thiole-5,6-diyl)-bis(azanediyl))-bis(methanylylidene)) (2*E*,2*E*')-bis (3-oxobutanoate)) (Scheme 1). The syntheses, crystal structures, electrochemistry, UV-vis-NIR spectroelectrochemistry, fluorescence and magnetic properties are described. This work demonstrates the possible interplay between SCO behaviour and fluorescence.

## Experimental Sections

### Synthesis

Synthesis of [Fe^II^L(TPPE)_0.5_] (**1**) We added 5 ml CH_2_Cl_2_ + 5 ml MeOH into the [Fe^II^L(MeOH)_2_] (20 mg, 0.03 mmol) and TPPE (10 mg, 0.015 mmol) mixture in the gloves box. After heating for 24 h at 70°C in an oven, the mixture was left undisturbed at room temperature. After one week, black rod-like crystals of **1** were gained. Yield: 12.7 mg (42%, grounded on TPPE). Anal. Calcd for C_47_H_38_FeN_4_O_4_S_6_: C 58.13, H 3.94, N 5.77%; found: C 58.00, H 3.80, N 5.61%.

Synthesis of [Fe^II^_3_L_3_(TPPE)_1.5_] (**2**) Compound **2** was synthesized by the same reactants as **1**, while after heating for 48 h at 70°C in an oven. It was left in an undisturbed place at room temperature. After one week, black block crystals of **2** were isolated. Yield: 18.8 mg (22%, grounded on TPPE). Anal. Calcd for C_141_H_113_Fe_3_N_12_O_12_S_18_: C 58.15, H 3.91, N 5.77%; found: C 57.98, H 3.75, N 5.59%.

## Result and Disscussion

### Structural Description

The crystals of **1** appropriate for X-ray structure characterization were obtained by hydrothermal method from precursor [Fe^II^L (MeOH)_2_] and bridging ligand TPPE at 70°C for 24 h. However, we could obtain **2** through lengthen the reaction time to 48 h.

**FIGURE 1:**
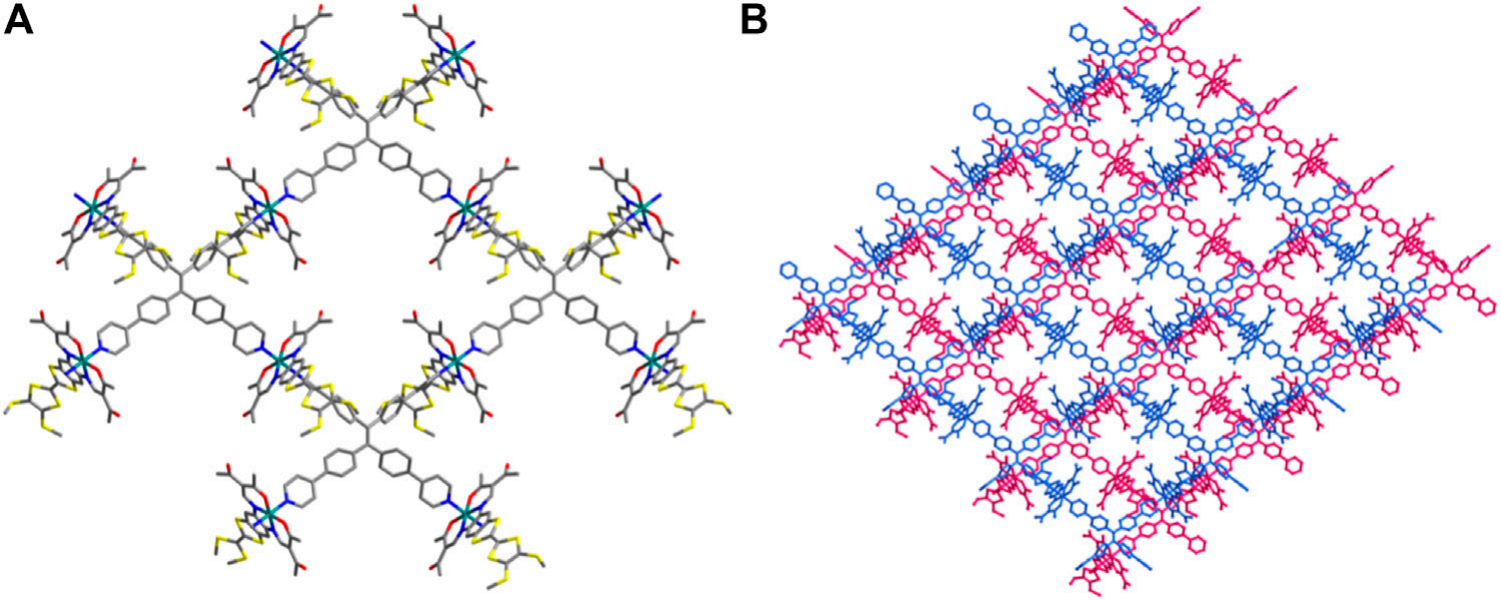
2D network **(A)** and adjacent interdigitated layers (two layers are shown with two different colours) along the a axis **(B)** for **1**. All of the hydrogen atoms are omitted for clarity, carbon - grey, nitrogen -blue, oxygen - red, sulfur - yellow, iron - teal.

The dark red needles of **1** [Fe^II^L (TPPE)_0.5_], crystallized in the monoclinic space group *C* 2/*c.* The unit cell involves one crystallographically independent [Fe^II^L] subunit, half a TPPE ligand (Supplementary Figure 1A). The Fe^II^ coordination is a slightly distorted [FeN_4_O_2_] octahedral coordination configuration in which two nitrogen atoms (N1 and N2) and two oxygen atoms (O1 and O2) from the TTF Schiff-base ligand constitute the basal plane, and two nitrogen atoms (N3 and N4) from tetradentate bridging TPPE (Supplementary Figure 1B), resulted in the expected square-grid [Fe^II^L (TPPE)_0.5_]. All of these donors (four nitrogen and two oxygen atoms) lie in *cis*-locations. The sum of angles among the basal atoms is close to 360°, indicating that Fe1, N1, N2, O1 and O2 share the same plane. The designed square-grid is the key feature of **1**, and the four diverging pyridines from the TPPE ligand are coordinated by the *trans* locations to the flat TTF Schiff-base ligand (Figure 1A). The formation of flat layer is caused by the rigid complanation of the TPPE ligand; and this phenomenon can avoid the penetration of layers, though there is vacant region between the squares. TTF Schiff-base ligand protrudes out of the layer such that adjacent layers are displaced, which resulted in a reduplicative part constituting of two layers per monoclinic *C* 2/*c* unit cell (Figure 1B). Typically, for SCO materials, crystallography methods could identify LS and HS states because of differences in bond lengths of the Fe^II^ centres between these two states (Δ = 0.14–0.24 Å) (Kahn, 1993). In the present case, the average coordination bond lengths are Fe-N_eq_ (1.882 Å), Fe-O_eq_ (1.930 Å) and Fe-N_ax_ (2.004 Å). Additionally, the angle for Fe1 [O_eq_-Fe1-O_eq_] is 89.1 (2)°. All these values for Fe1 are in agreement with those reported for Fe^II^ analogues with LS configurations (Kahn, 1993; Rodríguez-Jiménez et al., 2016; Wang et al., 2018b; Rosario-Amorin et al., 2018; Yuan et al., 2018; Schönfeld et al., 2020; Zappe et al., 2020)

**FIGURE 2:**
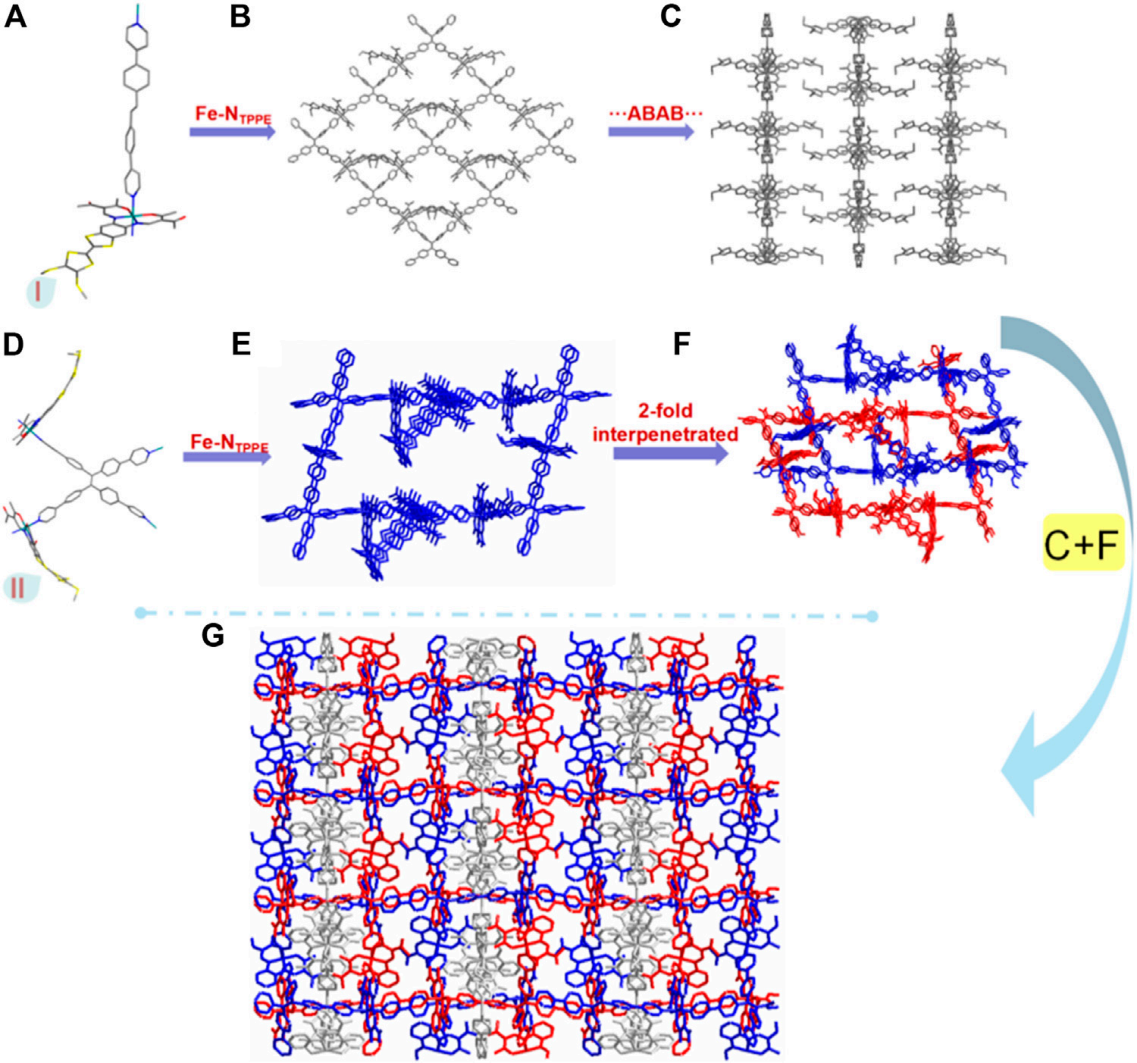
Monomer I **(A)**, a 2D network of monomer I **(B)**, the pacing structure of a 2D network of monomer I **(C)**, monomer II **(D)**, 3D frame structure of monomer II **(E)**, the 2-fold interpenetrated of 3D frame structure of monomer II **(F)** and the 3D interpenetrated framework including 2D network and 3D crystal structure along the ***a*** axis **(G)** for **2**. All of the hydrogen atoms are omitted for clarity, carbon - grey, nitrogen -blue, oxygen - red, sulfur - yellow, iron - teal.

With longer reaction time, a more compacted compound [(Fe^II^_3_L_3_ (TPPE)_1.5_)] (**2**), was obtained. It crystallizes in the monoclinic space group *C* 2/*c*. Three independent [Fe^II^L] and one and a half TPPE make up the unsymmetric part (Supplementary Figure 2A; Supplementary Table S1); each central Fe^II^ is six-coordinated in distorted octahedra where the two nitrogen atoms (N3 and N4A) from the bridging TPPE occupy the axial positions [angle [N3-Fe1-N4A 176.9 (3)°] and the other four atoms (N1, N2, O1 and O2) from the TTF Schiff-base ligand occupy the equatorial positions (sum of the trigonal angles is 358.3°) (Supplementary Figure 2B). The average angle of O_eq_-Fe-O_eq_ is 88.7° [O_eq_-Fe1-O_eq_ 88.5 (3)°, O_eq_-Fe2-O_eq_ 88.6 (2)° and O_eq_-Fe3-O_eq_ 88.9 (2)°] is similar to those found for LS Fe^II^ complexes (Kahn, 1993; Rodríguez-Jiménez et al., 2016; Wang et al., 2018b; Rosario-Amorin et al., 2018; Yuan et al., 2018; Schönfeld et al., 2020; Zappe et al., 2020). Additionally, the average bond lengths of Fe-N_eq_ is 1.891 Å [Fe1-N_eq_ 1.870 Å, Fe2-N_eq_ 1.890 Å and Fe3-N_eq_ 1.913 Å], Fe-O_eq_ is 1.930 Å [Fe1-O_eq_ 1.935 Å, Fe2-O_eq_ 1.932 Å and Fe3-O_eq_ 1.923 Å], and Fe-N_ax_ is 2.006 Å [Fe1-N_ax_ 1.993 Å, Fe2-N_ax_ 2.005 Å and Fe3-N_ax_ 2.021 Å], which is typical for LS Fe^II^ complexes (Kahn, 1993; Rodríguez-Jiménez et al., 2016; Wang et al., 2018b; Rosario-Amorin et al., 2018; Yuan et al., 2018; Schönfeld et al., 2020; Zappe et al., 2020). The TPPE and Fe1 ions form 2D layers stack in an ABAB fashion along the *c* axis (Figures 2A,C). The three-dimensional (3D) frameworks, containing TPPE, Fe2 and Fe3 ions, is 2-fold interpenetrated (Figures 2D–F). By virtue of the axial coordination and size of the TPPE, an unprecedented catenation of layers through two interpenetrated frameworks is formed (Figure 2G).

### Electrochemical Properties

**FIGURE 3:**
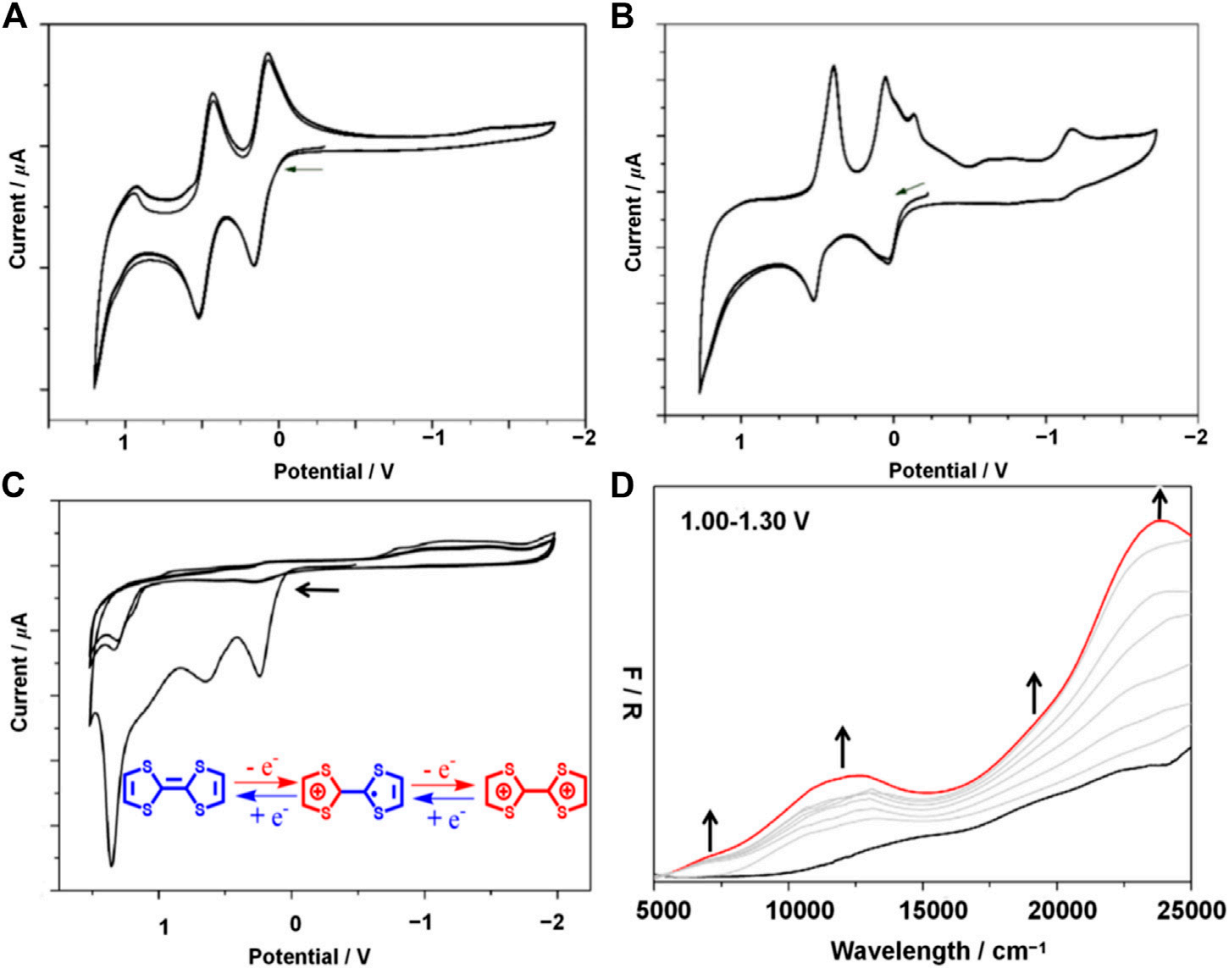
Solution state CVs of H_2_L measured over 3 consecutive sweeps **(A)**, solution state CVs of [Fe^Ⅱ^L(CH_3_OH)_2_] measured over 3 consecutive sweeps **(B)**, solid state CV for **1** obtained at 100 mVs^−1^ over 3 consecutive scans **(C)**, solid-state UV-vis-NIR spectroelectrochemistry of **1** at 0 V (black) and 1.30 V (red) **(D)**. Gray spectra correspond to the spectral transition over the applied potential range of 1.00–1.30 V. Experiment performed in 0.1 mol L^−1^ TBAPF_6_ in CH_2_Cl_2_. Arrows denote the direction of forward scan.

The cyclic voltammogram of H_2_L (Supplementary Figure 4) displayed two highly invertible oxidation waves at E_1/2_ = 0.11 and 0.47 V *vs* Fc/Fc^+^ (compared to TTF^0^/TTF^·+^ = −0.06 and TTF^·+^/TTF^2+^ = 0.38 V of TTF itself) (Canevet et al., 2009; Narayan et al., 2012; Su et al., 2017), indicating the construction of the TTF^·+^ and TTF^2+^, respectively. Very little change is seen in the CV collected over multiple sweeps (Figure 3A) which demonstrates the stability of the Schiff-base-like ligand to redox change. At faster scan rates (Supplementary Figure 5), a third quasi-reversible oxidation wave was found at 0.99 V, which may tentatively be ascribed to oxidation of the macrocycle i.e. proton assisted oxidation of the ketone groups.

Upon complexation of H_2_L with Fe^2+^ ([Fe^Ⅱ^L(CH_3_OH)_2_]) additional redox features were observed (Supplementary Figure 4). Two overlapping invertible oxidation waves were observed at −0.05 and 0.08 V which may be due to the Fe^2+^/^3+^ and TTF^0^/TTF^·+^ redox couples, respectively. A third, reversible oxidation peak found at 0.46 V corresponds to the oxidation of the TTF^·+^ cation to TTF^2+^. The redox potentials of the latter two oxidation waves coincide well with the free base which suggests that coordination of the macrocycle to Fe^2+^ does not significantly affect the electronic distribution at the TTF site; thus minimal electron delocalization is expected to occur between the TTF and amine functionalities across the aryl spacer. Over multiple potential sweeps, the CVs obtained overlapped well, which confirms the stability of [Fe^Ⅱ^L(CH_3_OH)_2_] with redox manipulation (Figure 3B).

The electrochemical data for **1** was quite different to that of the free base H_2_L and discrete complex [Fe^Ⅱ^L(CH_3_OH)_2_] likely to be a result of the crystalline packed nature of the coordination polymer. The CV (Supplementary Figure 7) reveals four irreversible oxidation processes at E_onset_ = 0.17, 0.65, 1.01 and 1.29 V which may be assigned to the construction of a TTF^0^/TTF^·+^, (TTF^·+^)_2_, TTF^·+^/TTF^2+^ and (TTF^2+^)_2_, respectively. Thus, stacking interactions of TTF moieties between interpenetrated nets may stabilise the formation of mixed-valence species. The irreversible nature of these redox processes, however, suggests either that the framework material is unstable to these manipulations or rather that an irreversible structural change occurs in response to the change in oxidation state. This was confirmed with cycling experiments where, upon the second sweep, the current associated with each aforementioned process significantly decreases (Figure 3C). Multiple overlapping reduction processes were observed between −0.86 and −1.50 V which were also apparent in the CV of [Fe^Ⅱ^L(CH_3_OH)_2_]. These features may thus be assigned to the discrete complex unit, however, the origin of these processes remains elusive.

We have took the high complexity of CV and square wave voltammetry into consideration and performed the UV-vis-NIR spectroelectrochemistry (SEC) electrochemical processes in order to examine the electrochemical processes electrochemical processes (Figure 3D; Supplementary Figures 10-11). The SEC data revealed a marked spectral change at 1.00 V; new low energy features were observed at *ca.* 7,000 and 12,000 cm^−1^ as well as an intensification of the bands at 19,000 and 24,000 cm^−1^ which are owing to the construction of the TTF^•+^ (Canevet et al., 2009). The observation of these bands in the as-synthesized material suggests the presence of TTF^•+^ may be as result of the relatively low oxidation potential associated with the TTF^0/•+^ redox couple (0.17 *vs* Fc/Fc^+^ determined by CV).

### Magnetic Properties

**FIGURE 4:**
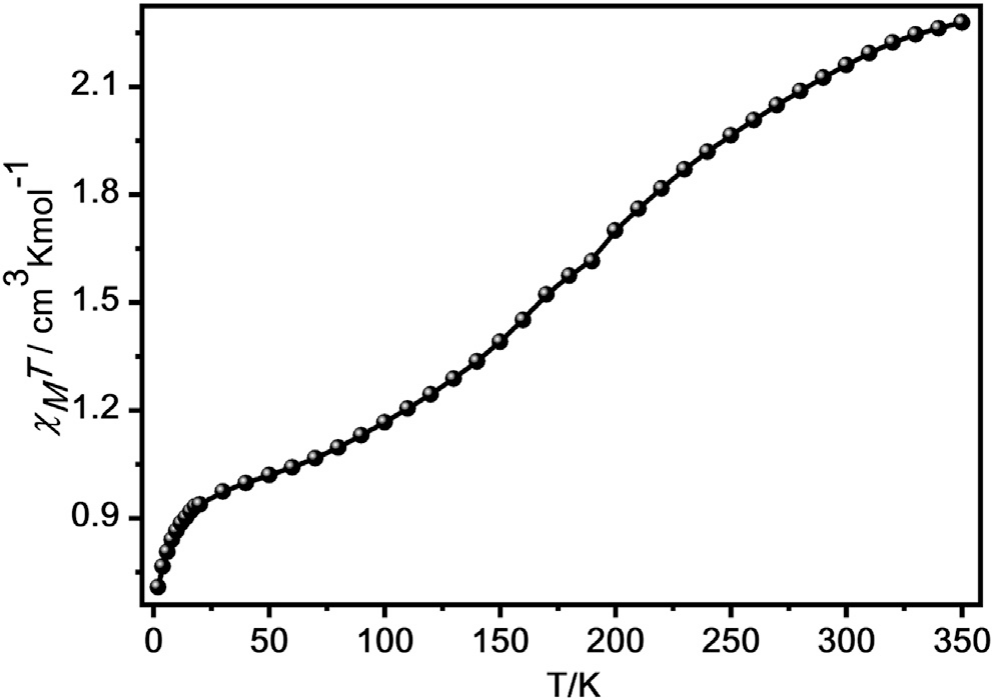
The temperature-dependent of χ_*M*_ *T* product in 1,000 Oe for **1**.

We investigated the magnetic properties for **1** and **2** from 2 to 350 K in 1,000 Oe (Figure 4; Supplementary Figure 12). The χ_*M*_ *T* product at 350 K of **1** was 2.28 cm^3^ K mol^−1^ (Figure 4), which indicates that there were about 70% transformation from [LS-LS] to [HS-HS] (Kahn, 1993; Rodríguez-Jiménez et al., 2016; Wang et al., 2018b; Rosario-Amorin et al., 2018; Yuan et al., 2018; Schönfeld et al., 2020; Zappe et al., 2020). As the temperature reduced, the χ_*M*_ *T* value gradually decreased and achieved a plateau product of 1.13 cm^3^ K mol^−1^ at 90 K, indicating the spin transition from HS state of Fe^II^ center to its LS state (Kahn, 1993; Rodríguez-Jiménez et al., 2016; Rosario-Amorin et al., 2018; Wang et al., 2018b; Yuan et al., 2018; Schönfeld et al., 2020; Zappe et al., 2020). Below 28 K, the χ_*M*_ *T* product sharply decreases, attaining 0.71 cm^3^ K mol^−1^ at 2 K, which can be owing to the presence of zero-field splitting of the residual HS Fe^II^ ions or antiferromagnetic interaction among Fe^II^ centres (Suleimanov et al., 2015). However, at 350 K, the χ_*M*_ *T* product for **2** is 2.16 cm^3^ K mol^−1^, which is much lower than the expected product for three isolated HS Fe^II^ centres (Supplementary Figure 12), indicating that the LS Fe^II^ centres are dominant in **2** (Garcia et al., 2011; Wang et al., 2015; Schönfeld et al., 2020; Zappe et al., 2020).

### Fluorescence Properties

The fluorescence emission of **1** at room temperature is compared to that of the pure TPPE (Supplementary Figure 13). TPPE itself displays a strong emission at 472 nm when excited at 360 nm (Supplementary Figure 13). Under the identical excitation wavelength, the emission bands for **1** and **2** occur at 442 and 464 nm, and the fluorescence intensities for **1** and **2** were reduced by a factor of three, respectively (Supplementary Figure 13). The prominent hypochromic effect occured in fluorescence spectra, which may be impacted by the π-π* conversion in the centre of ligand. And these results may be attributed to the coordination between Schiff-base ligand and the metal centre as well as the introduction of TPPE to the Fe^II^ complex (Ge et al., 2019).

**FIGURE 5:**
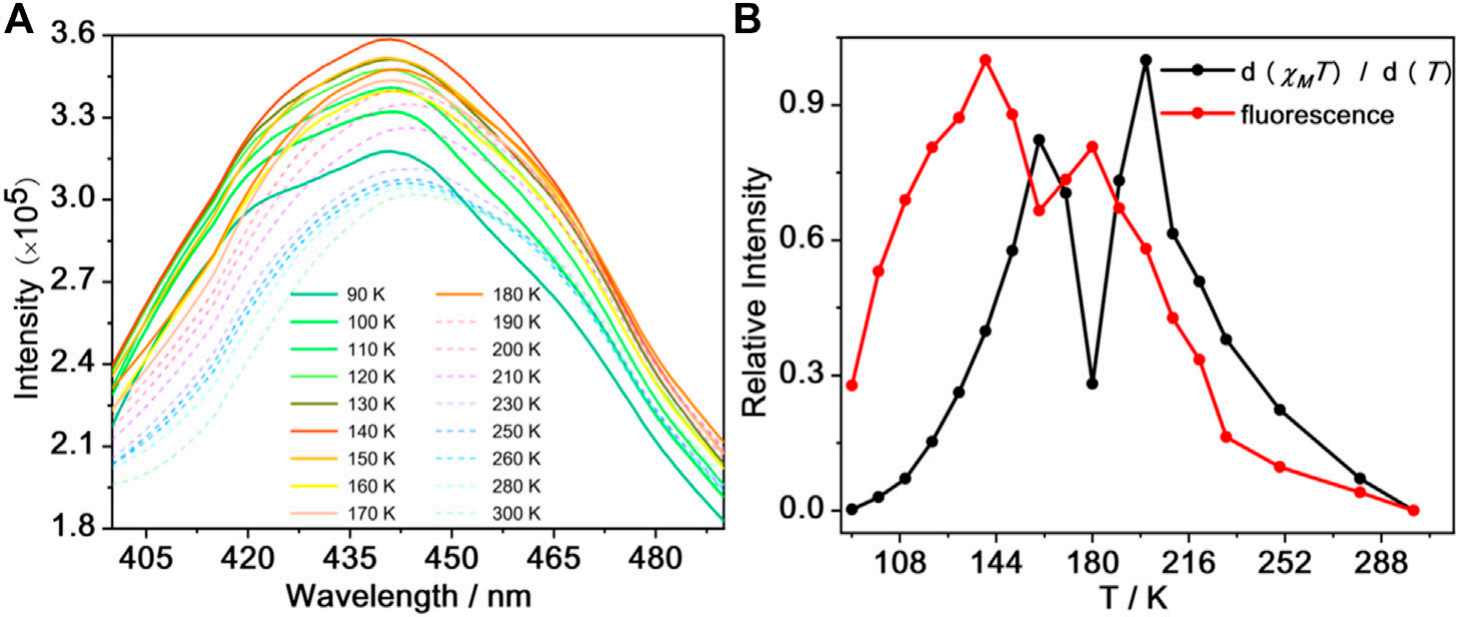
The fluorescence spectra for **1** over the temperature range 90–300 K **(A)** and the *d* (χ_*M*_T)/*d*(T) curve (black line) and fluorescence normalized curve (red line) for **1** **(B)**.

In search for the connection between SCO and fluorescence properties in **1**, a study of its fluorescence emission was investigated while varying the temperature from 90 to 300 K (Figure 5A; Supplementary Figure 14). Upon warming, the fluorescence intensity of **1** increased gradually, reaching a maximum at 140 K and the fluorescence intensity decreased until 160 K. However, when the temperature increased to 160 K, the fluorescence intensity began to increase suddenly until 180 K, followed by a decrease in intensity upon further warming. These drastic temperature-dependent variations in fluorescence intensity occur in the range 90–300 K which agrees moderately well with the thermally induced SCO behavior of **1**. We speculate that the changes in coordination geometry and bond lengths between the ligand and Fe^II^ ions associated with the spin transition could affect the fluorescence properties mentioned before (Garcia et al., 2011; Wang et al., 2015).

To further check on this hypothesis, we have made normalized data of *d* (*χ*_*M*_T)/*d*T and fluorescence intensity of **1** in the 90–300 K temperature region (warming mode) (Figure 5B). From this plot, the SCO transition profile of compound **1** is consistent with the change in fluorescence intensity, suggesting that the fluorescence change is related to the SCO transition; the slight difference between the peaks in Figure 5B is likely due to differences in the thermal sweep rates of the two experiments. We speculate that the main reason is the invertible electron transport between the antibonding orbitals of Fe^II^ ions and the lowest unoccupied molecular orbital of TPPE (Wang et al., 2015; Ge et al., 2019). For comparison, the temperature dependence fluorescence spectra of the precursor [Fe^II^L(MeOH)_2_] was measured. It exhibits weaker fluorescence signal under excitation at 360 nm from 90 to 280 K (Supplementary Figure 15). The emission intensity gradually increased from 90 to 260 K and it decreased from 260 to 280 K at about 435 nm, which may be attributed to the vibration of molecular geometry. In conclusions, the drastic temperature-dependent variations in emission intensity maybe assigned to the coordination of the N atom from bridging ligand and the central Fe^II^ ion from precursor [Fe^II^L(MeOH)_2_]. It can be evidenced that the almost monotone decreasing in the fluorescence intensity of TPPE can make clear the electron transport mechanism demonstrated above.

## Conclusion

In summary, two Fe^II^ coordination polymers [Fe^II^L (TPPE)_0.5_] **1**) and [(Fe^II^_3_L_3_ (TPPE)_1.5_)] **2**) have been successfully prepared by introducing the redox-active TTF unit as well as the fluorescent TPPE ligand. Magnetic investigations reveal that **1** exhibits SCO behaviour, while **2** remains in the LS state. Because of the synergetic effect between SCO and fluorescence, the changes of the spin state of complex **1** could regulate the luminescence intensity of the TPPE ligand. Moreover, the electrochemical studies show that these coordination polymers reserved the redox activity of the TTF unit. Further efforts aimed towards the preparation of diverse multifunctional SCO materials exhibiting higher transition temperature show great promise and are currently being undertaken in our laboratory.

## Data Availability

All datasets generated for this study are included in the article/Supplementary Material.
