# Supplementary material for: Iron(II) Spin Crossover Coordination Polymers Derived From a Redox Active Equatorial Tetrathiafulvalene Schiff-Base Ligand
Source: Front Chem. 2021 Aug 2;9:692939. doi: 10.3389/fchem.2021.692939 (PMC8365465; doi:10.3389/fchem.2021.692939)
Supplement: Supplementary file 1 [file DataSheet2.PDF]

Supplementary Material for

Iron(II) Spin Crossover Coordination Polymers derived from a Redox Active Equatorial Tetrathiafulvalene Schiff-base Ligand

Ya-Ru Qiu[1,2], Long Cui[2], Jing-Yuan Ge[3*], Mohamedally Kurmoo[2,4*], Guijun Ma[1*] and Jian Su[2*]

[1]*School of Physical Science and Technology, ShanghaiTech University, Shanghai 201210, P. R. China*

[2]*State Key Laboratory of Coordination Chemistry, School of Chemistry and Chemical Engineering, Nanjing National Laboratory of Microstructures, Nanjing University, Nanjing 210023, P. R. China*

[3]*College of Chemistry & Materials Engineering, Wenzhou University, Wenzhou 325035, P. R. China*

[4]*Institut de Chimie de Strasbourg, CNRS-UMR 7177 Université de Strasbourg 4 rue Blaise Pascal, 67008 Strasbourg, France*

.

Contents

1. **Experimental Sections**

2. **Syntheses**

3. **X-ray Crystallography**

Table S1 Crystallographic data and structure refinement parameters for **1** and **2**

Table S2. Crystallographic data and structure refinement parameters for **H_2L** and **$[Fe^{II}L(CH_3OH)_2]$**.

Table S3. Selected bond lengths (Å) and angles (°) for **1**.

Table S4. Selected bond lengths (Å) and angles (°) for **2**.

Table S5. Selected bond lengths (Å) and angles (°) for **H_2L**.

Table S6. Selected bond lengths (Å) and angles (°) for **$[Fe^{II}L(CH_3OH)_2]$**.

Supplementary Figure 1. Crystal structure for **1**.

Supplementary Figure 2. Crystal structure for **2**.

Supplementary Figure 3. Crystal structure for **H_2L** and **$[Fe^{II}L(CH_3OH)_2]$**.

4. **Characterizations**

4.1 Electrochemical Properties for **H_2L**, **$[Fe^{II}L(CH_3OH)_2]$**, **1** and **2**.

4.2 Spectroelectrochemistry properties for **1**.

4.3 Magnetic properties for **2**.

4.4 Fluorescence properties for TPPE, **1** and **2**.

4.5 The ^{1}H NMR spectrum for **H_2L**.

5. **References**

1. Experimental Sections

1.1 Physical Measurements

^1H NMR spectra were recorded on a Bruker DPX 400 MHz spectrometer with internal standard tetramethylsilane (TMS). Magnetic susceptibilities were performed with a Quantum Design MPMS-SQUID-VSM magnetometer in the temperature range from 2 K to 350 K and an applied field of 1 kOe. Pascal's constants and an experimental correction for the sample holder was applied to caculate diamagnetic corrections. Solid-state cyclic voltammetry (CV) was carried out using a BASi Epsilon Electrochemical Analyzer with ferrocene (Fc) as an internal reference. The measurement condition: Ar atmosphere, a three-electrode configuration (the counter electrode is a Pt wire and the reference electrode is an Ag/AgCl), A the electrolyte is a 0.1 M tetrabutylammonium hexafluorophosphate ([*n*-$Bu_4N]PF_6/CH_3CN$), scan rate of 20-500 mV/s. UV-vis-NIR spectra were obtained on the samples at room temperature using a CARY5000 spectrophotometer equipped with a Harrick Praying Mantis accessory over the wavenumber range 5000-40000 cm^{-1}. $BaSO_4$ was used to acquire the baseline spectrum. Spectra are reported as the Kubelka-Munk transform, where $F(R) = (1-R)^2/2R$ (R is the diffuse reflectance of the sample compared to $BaSO_4$). Temperature-dependent fluorescence spectra were recorded using a Horiba FluoroMax-4 spectrofluorometer with slit width set as 5/5 nm and a custom built vacuum vessel equipped with a cryostat.

1.2 Materials and Syntheses

The reagents and solvents were commercially available and were used as received. Dichloromethane (CH_2Cl_2), methanol (MeOH) and acetonitrile (CH_3CN) were all degassed prior to use. H_2L (H_2L = 2,2'-(((2-(4,5-bis(methylthio)-1,3-dithiol-2-ylidene)benzo [*d*][1,3]dithiole-5,6-diyl)-bis(azanediyl))-bis(methanylylidene))(2*E*,2*E*')-bis(3-oxobutanoate) and TPPE (TPPE = 1,1,2,2-tetrakis(4-(pyridine-4-yl)phenyl)-ethene) were prepared according to literature methods (1, 2).

2. Syntheses

Synthesis of H_2L. 5,6-Diamino-2-(4,5-bis(propylthio)-1,3-dithio-2-ylidene)-benzo[*d*]- 1,3-dithiole (376 mg, 1.0 mmol), ethoxymethyleneacetylacetone (312 mg, 2.0 mmol) dissolved in 15 mL ethanol was heated to reflux for 2 h. Crystals suitable for the single-crystal X-ray analysis were

obtained by slow evaporation of the CH_2Cl_2/CH_3CN solution of **H_2L**. Yield: 447 mg (75%). ^1H NMR (400 MHz, $CDCl_3$, ppm): δ 12.95 (d, *J* = 11.6 Hz, 2H), 8.02 (d, *J* = 11.7 Hz, 2H), 7.12 (s, 2H), 2.56 (s, 6H), 2.45 (s, 6H), 2.38 (s, 6H).

Synthesis of [$Fe^{II}L(MeOH)_2$]. **H_2L** (120 mg, 0.2 mmol) and anhydrous ferrous acetate (52 mg, 0.3 mmol) were dissolved in 16 mL CH_2Cl_2/MeOH (3:1, v/v). The dark brown suspension was stirred in the N_2 atmosphere. After stirring 12 h, the resulting dark green precipitate was filtered and washed with MeOH. The slow evaporation of [$Fe^{II}L(MeOH)_2$] in DCM/MeOH yielded black block crystals. Yield: 170 mg (81%, based on H_2L).

3. X-ray Crystallography

The single crystals of **1**, **2**, **H_2L** and **[$Fe^{II}L(CH_3OH)_2$]** were determined with a Bruker Smart Apex II CCD diffractometer at 123(2) K for **1** and **2** , and 296(2) K for **H_2L** and **[$Fe^{II}(L)(CH_3OH)_2$]** (MoKα radiation, λ = 0.71073 Å). The raw data were integrated into SHELX-format reflection files and corrected for Lorentz and polarization effects using SAINT (3). Corrections for incident and diffracted beam absorption effects were applied using SADABS (4). No change of diffraction intensity was evidenced when data collected. The structures were solved and refined against F^2 by the full-matrix least-squares using the SHELXL-2016/6 program (5). All non-hydrogen atoms were refined with anisotropic thermal parameters, and hydrogen atoms of the organic ligands were calculated theoretically onto the specific atoms and refined isotropically with fixed thermal factors. The crystal data, data collection parameters, and refinement statistics of **1**, **2**, **H_2L** and **[$Fe^{II}L(CH_3OH)_2$]** are provided in **Tables S1** and **S2,** respectively. Relevant interatomic bond distances and bond angles are given in **Tables S3-S6**. CCDC reference numbers: 983310 **(1)**, 983311 **(2),** 2026386 **(H_2L)**, 2026387 **([$Fe^{II}L(CH_3OH)_2$])**.

Table S1 Crystallographic data and structure refinement parameters for **1** and **2**.

**Complexes**	**1**	**2**
Formula	$C_{47}H_{38}FeN_4O_4S_6$	$C_{141}H_{113}Fe_3N_{12}O_{12}S_{18}$
Mr	971.02	2912.06
Wavelength, Å	0.71073	0.71073

Crystal system	monoclinic	monoclinic
Space group	*C* 2/*c*	*C* 2/*c*
a, Å	34.962(4)	34.040(5)
b, Å	25.670(3)	27.578(3)
c, Å	18.2005(19)	43.476(5)
β, °	120.501(2)	112.554(3)
V, Å^3	14074(3)	37692(8)
Z	8	8
d_{calc}, g/cm^3	0.917	1.026
μ, mm^{-1}	0.42	0.48
F(000)	4016	12040
Reflections collected	39644	80809
Unique reflections	12389	33131
GOF	1.17	1.07
$R_1{}^a$, $wR_2{}^b$ [I > 2σ(I)]	0.0871, 0.1779	0.0929, 0.1833
$R_1{}^a$, $wR_2{}^b$ [all data]	0.1386, 0.1991	0.1596, 0.2165

$^aR_1 = \sum||F_o| - |F_c||/\sum|F_o|$ $^bwR_2 = \{\sum[w(F_o^2 - F_c^2)_2]/\sum[w(F_o^2)^2]\}^{1/2}$.

Table S2. Crystallographic data and structure refinement parameters of **H_2L** and **[$Fe^{II}L(CH_3OH)_2$]**.

**Complexes**	**H_2L**	**[$Fe^{II}L(CH_3OH)_2$]**

Formula	$C_{26}H_{27}N_3O_4S_6$	$C_{30}H_{46}FeN_2O_{10}S_6$
Mr	637.87	842.90
Wavelength, Å	0.71073	0.71073
Crystal system	monoclinic	monoclinic
Space group	$P2_1/n$	$P2_1/c$
a, Å	5.0923 (5)	15.835 (5)
b, Å	15.4249 (17)	28.144 (9)
c, Å	38.309 (4)	8.961 (3)
β, °	91.071 (3)	100.929
V, $Å^3$	3008.6 (5)	3921 (2)
Z	4	4
d_{calc}, g/cm^3	1.408	1.428
μ, mm^{-1}	0.49	0.76
F(000)	1328	1768
Reflections collected	26381	23695
Unique reflections	7005	7984
Goodness-of-fit on F^2	1.10	1.04
$R_1{}^a$, $wR_2{}^b$ [I > 2σ(I)]	0.0882, 0.1662	0.0749, 0.1900
$R_1{}^a$, $wR_2{}^b$ [all data]	0.1525, 0.1872	0.1092, 0.2101

$^aR_1 = \sum||F_o| - |F_c||/\sum|F_o|$ $^bwR_2 =\{\sum[w(F_o^2 - F_c^2)_2]/ \sum[w(F_o^2)^2]\}^{1/2}$.

Table S3. Selected bond lengths (Å) and angles (°) for **1.**

Complex **1**			
Fe1—N1	1.876 (5)	N1—Fe1—N2	84.7 (2)
Fe1—N2	1.887 (5)	N1—Fe1—O1	93.5 (2)
Fe1—O1	1.924 (4)	N2—Fe1—O1	178.1 (2)
Fe1—O2	1.936 (5)	N1—Fe1—O2	176.3 (2)
Fe1—N3	2.000 (4)	N2—Fe1—O2	92.7 (2)
Fe1—N4^{ii}	2.008 (4)	O1—Fe1—O2	89.1 (2)
N4—Fe1^{iii}	2.008 (4)	N1—Fe1—N3	90.35 (19)
N1—Fe1—N4^{ii}	92.76 (19)	N2—Fe1—N3	92.60 (19)
N2—Fe1—N4^{ii}	90.58 (19)	O1—Fe1—N3	88.06 (18)
O1—Fe1—N4^{ii}	88.86 (18)	O2—Fe1—N3	87.04 (18)
O2—Fe1—N4^{ii}	89.99 (18)	N3—Fe1—N4^{ii}	175.7 (2)

Symmetry codes: (i) -x+1, y, -z+1/2; (ii) x+1/2, y+1/2, z+1; (iii) x-1/2, y-1/2, z-1.

Table S4. Selected bond lengths (Å) and angles (°) for **2.**

Complex **2**			
Fe1—N1	1.846 (7)	N1—Fe1—N2	85.8 (3)
Fe1—N2	1.894 (7)	N1—Fe1—O1	178.4 (3)
Fe1—O1	1.927 (6)	N2—Fe1—O1	92.7 (3)
Fe1—O2	1.943 (6)	N1—Fe1—O2	93.0 (3)
Fe1—N3	1.991 (6)	N2—Fe1—O2	178.8 (3)
Fe1—N4^{ii}	1.994 (5)	O1—Fe1—O2	88.5 (3)
Fe2—N6	1.889 (5)	N1—Fe1—N3	89.4 (3)
Fe2—N5	1.891 (6)	N2—Fe1—N3	90.3 (3)
Fe2—O5	1.930 (5)	O1—Fe1—N3	91.2 (2)
Fe2—O6	1.933 (5)	O2—Fe1—N3	89.5 (2)
Fe2—N7	2.003 (5)	N1—Fe1—N4^{ii}	90.8 (2)
Fe2—N9^{iii}	2.007 (5)	N2—Fe1—N4^{ii}	92.9 (2)
Fe3—N12	1.904 (6)	O1—Fe1—N4^{ii}	88.6 (2)
Fe3—N11	1.921 (5)	O2—Fe1—N4^{ii}	87.4 (2)
Fe3—O10	1.921 (4)	N3—Fe1—N4^{ii}	176.9 (3)
Fe3—O9	1.925 (5)	N6—Fe2—N5	85.5 (2)
Fe3—N8^{iv}	2.010 (6)	N6—Fe2—O5	178.0 (2)
Fe3—N10	2.032 (5)	N5—Fe2—O5	92.7 (2)
N4—Fe1^{v}	1.994 (5)	N6—Fe2—O6	93.2 (2)

N8—Fe3^{vi}	2.010 (6)	N5—Fe2—O6	178.5 (2)
N9—Fe2^{vii}	2.007 (5)	O5—Fe2—O6	88.6 (2)
N12—Fe3—O9	177.4 (2)	N6—Fe2—N7	90.0 (2)
N11—Fe3—O9	92.7 (2)	N5—Fe2—N7	90.0 (2)
O10—Fe3—O9	88.9 (2)	O5—Fe2—N7	90.75 (19)
N12—Fe3—N8^{iv}	91.1 (2)	O6—Fe2—N7	90.8 (2)
N11—Fe3—N8^{iv}	91.5 (2)	N6—Fe2—N9^{iii}	89.7 (2)
O10—Fe3—N8^{iv}	87.4 (2)	N5—Fe2—N9^{iii}	91.9 (2)
O9—Fe3—N8^{iv}	87.2 (2)	O5—Fe2—N9^{iii}	89.62 (19)
N12—Fe3—N10	93.9 (2)	O6—Fe2—N9^{iii}	87.3 (2)
N11—Fe3—N10	93.4 (2)	N7—Fe2—N9^{iii}	178.0 (2)
O10—Fe3—N10	87.9 (2)	N12—Fe3—N11	85.3 (2)
O9—Fe3—N10	87.9 (2)	N12—Fe3—O10	93.0 (2)
N8^{iv}—Fe3—N10	173.3 (2)	N11—Fe3—O10	177.9 (2)

Symmetry codes: (i) -x+1, y, -z+3/2; (ii) x+1/2, y+1/2, z; (iii) x+1/2, -y+3/2, z+1/2; (iv) x+1/2, y-1/2, z; (v) x-1/2, y-1/2, z; (vi) x-1/2, y+1/2, z; (vii) x-1/2, -y+3/2, z-1/2.

Table S5. Selected bond lengths (Å) and angles (°) for **H_2L.**

**H_2L**			
S1—C10	1.761 (4)	S6—C21	1.753 (4)
S1—C19	1.766 (4)	S6—C22	1.777 (6)
S2—C9	1.749 (4)	C19—C20	1.330 (6)
S2—C19	1.757 (4)	C10—S1—C19	95.23 (19)
S3—C20	1.752 (4)	C9—S2—C19	95.3 (2)
S3—C23	1.755 (4)	C20—S3—C23	95.6 (2)
S4—C20	1.759 (4)	C20—S4—C21	94.8 (2)
S4—C21	1.759 (4)	C23—S5—C24	103.4 (3)
S5—C23	1.748 (4)	C21—S6—C22	101.9 (3)
S5—C24	1.767 (6)		

Table S6. Selected bond lengths (Å) and angles (°) for **$[Fe^{II}L(CH_3OH)_2]$.**

**$[Fe^{II}L(CH_3OH)_2]$**			
Fe1—O3	2.003 (4)	O2—Fe1—N2	164.09 (16)

Fe1—O2	2.008 (4)	N1—Fe1—N2	79.01 (16)
Fe1—N1	2.094 (4)	O3—Fe1—O6	89.99 (15)
Fe1—N2	2.097 (4)	O2—Fe1—O6	87.21 (15)
Fe1—O6	2.165 (4)	N1—Fe1—O6	94.63 (15)
Fe1—O5	2.232 (4)	N2—Fe1—O6	98.82 (16)
C19—C20	1.340 (7)	O3—Fe1—O5	86.29 (15)
O3—Fe1—O2	109.51 (15)	O2—Fe1—O5	86.69 (15)
O3—Fe1—N1	164.17 (16)	N1—Fe1—O5	91.02 (15)
O2—Fe1—N1	85.87 (15)	N2—Fe1—O5	88.65 (16)
O3—Fe1—N2	85.33 (16)	O6—Fe1—O5	171.36 (14)

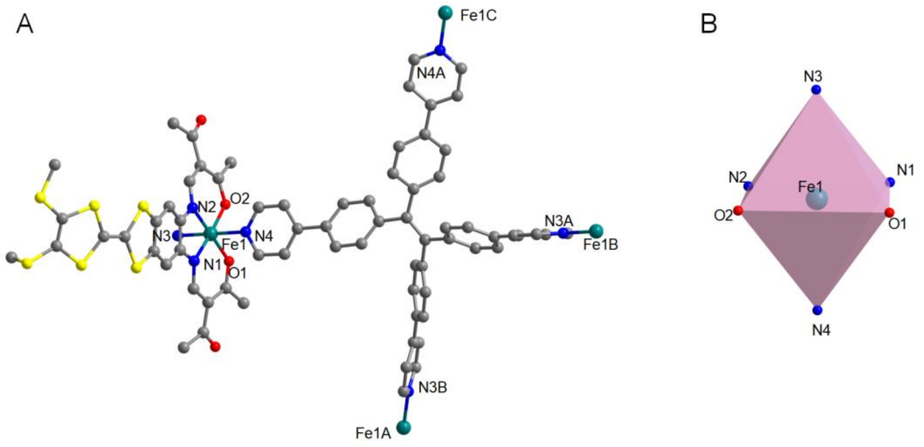

Supplementary Figure 1. Coordination environment around the Fe^{II} centre (A), the octahedron coordination configuration of the Fe^{II} centre (B) for **1**. All of the hydrogen atoms are omitted for clarity, carbon - grey, nitrogen -blue, oxygen - red, sulphur - yellow, iron - teal.

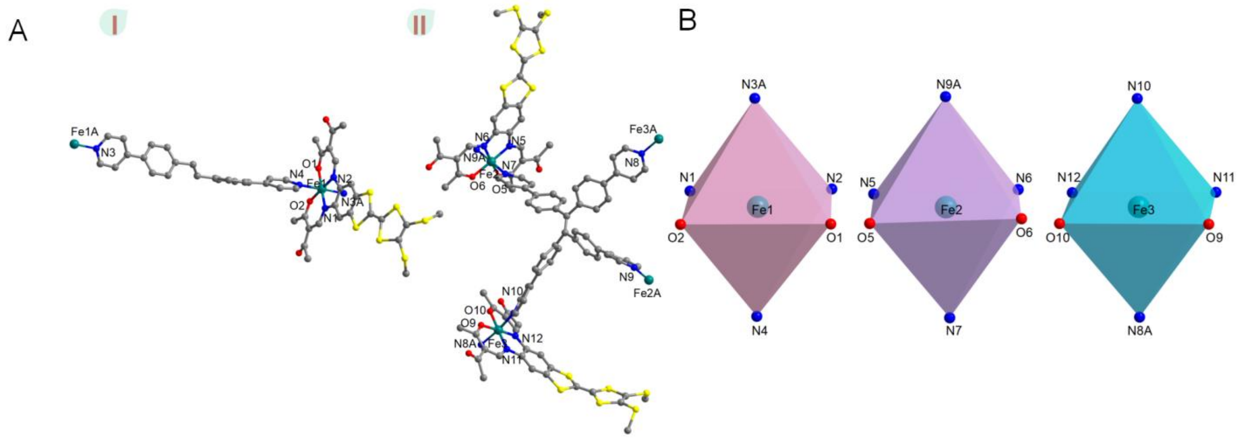

Supplementary Figure 2. Coordination environment around the Fe^{II} centre (A), the octahedron coordination configuration of the Fe^{II} centre (B) for **2**. All of the hydrogen atoms are omitted for clarity, carbon - grey, nitrogen -blue, oxygen - red, sulphur - yellow, iron - teal.

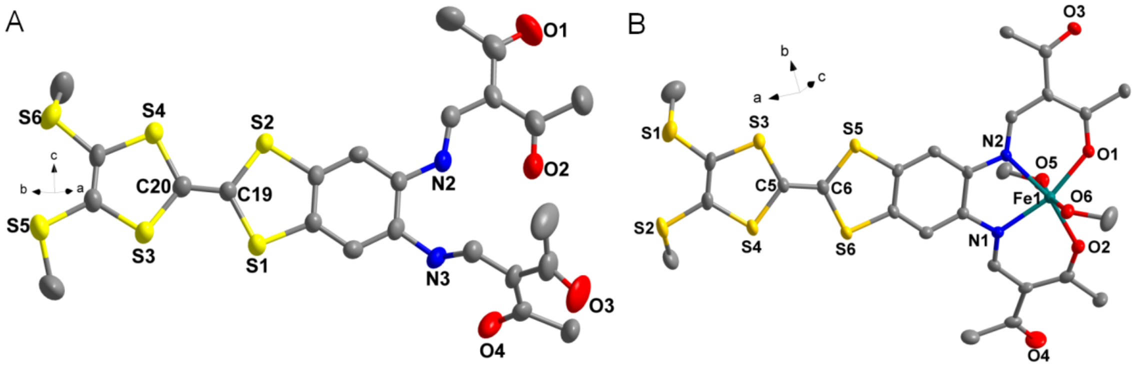

Supplementary Figure 3. Crystal structure for $\mathbf{H_2L}$ (A) and $\mathbf{[Fe^{II}L(CH_3OH)_2]}$ (B). All of the hydrogen atoms are omitted for clarity, carbon - grey, nitrogen -blue, oxygen - red, sulphur - yellow, iron - teal.

4. Characterizations

4.1 Electrochemical Properties for $\mathbf{H_2L}$, $\mathbf{[Fe^{II}L(CH_3OH)_2]}$, **1** and **2**.

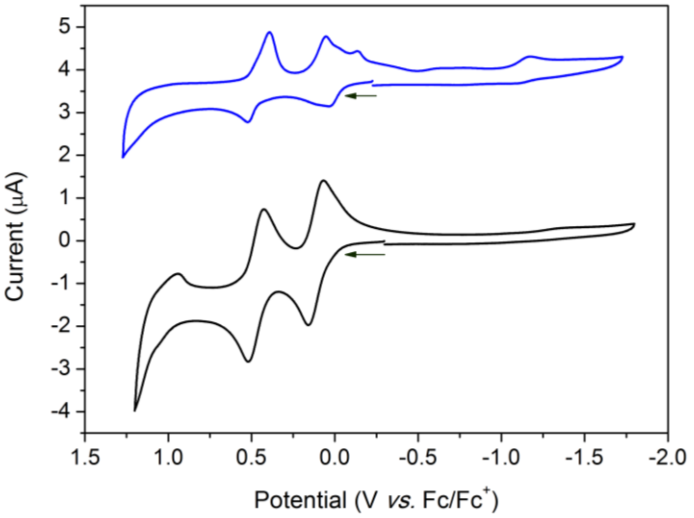

Supplementary Figure 4. Solution state CVs of **H_2L** (black) and **[$Fe^{II}L(CH_3OH)_2$]** (blue) measured at 100 mVs^{-1}. Experiment performed in 0.1 M $TBAPF_6$ in CH_2Cl_2 supporting electrolyte. Arrows indicate the direction of forward scan.

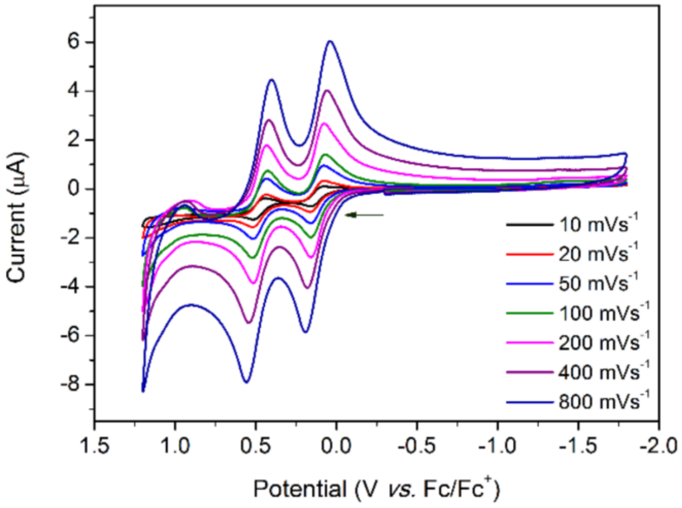

Supplementary Figure 5. Solution state CVs of **H_2L** measured over multiple scan rates. Experiment performed in 0.1 M $TBAPF_6$ in CH_2Cl2 supporting electrolyte. Arrow indicates the direction of forward scan.

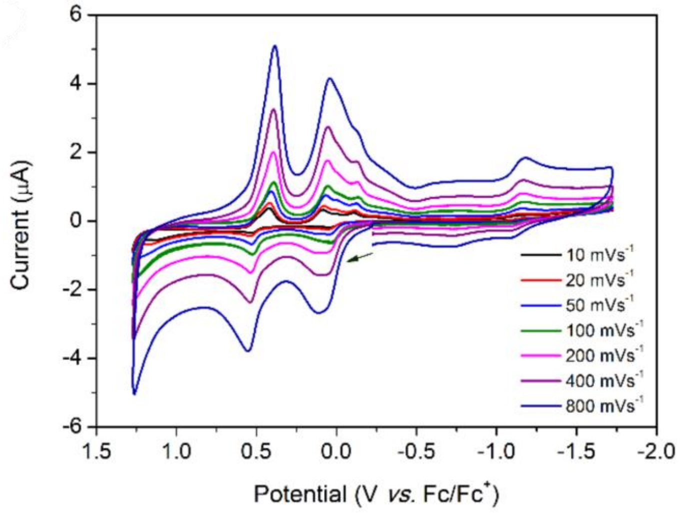

Supplementary Figure 6. Solution state CVs of **[$Fe^{II}L(CH_3OH)_2$]** measured over multiple scan rates. Experiment performed in 0.1 M $TBAPF_6$ in CH_2Cl_2 supporting electrolyte. Arrows indicate the direction of forward scan.

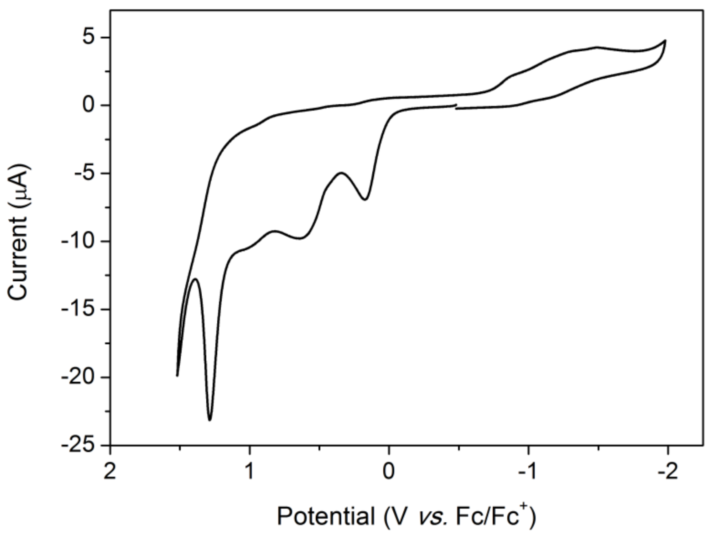

Supplementary Figure 7. Solid state CV for **1** obtained at 100 m Vs^{-1} in 0.1 M $TBAPF_6$ in CH_3CN supporting electrolyte. Arrow indicates the direction of forward scan.

Owing to their similar components and bonding, the electrochemical behaviors of **1** and **2** also demonstrate strong similarities. The anodic region of the CV of **2** (**Supplementary Figure 8**) exhibits four irreversible oxidation processes at onset potentials of 0.24, 0.65, 1.09 and 1.36 V, where the latter two are assigned to the formation of the radical cation and dication states of the TTF ligand, respectively. Upon cycling, these features similarly diminish in current, likely due to degradation of the framework or irreversible structural rearrangements. The cathodic region also demonstrates broad overlapping features in the range -0.80 to -1.51 V which may be tentatively ascribed to processes at the Fe^{II} complex unit.

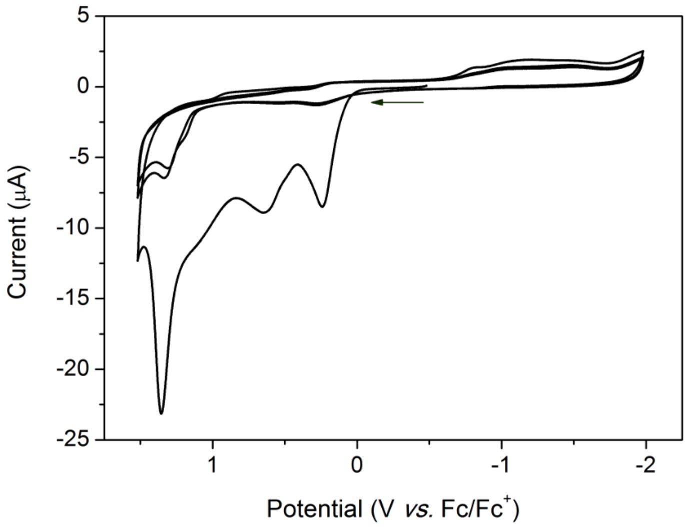

Supplementary Figure 8. Solid state CV for **2** obtained at 100 mVs^{-1} over 3 consecutive scans. Experiment performed in 0.1 M $TBAPF_6$ in CH_3CN supporting electrolyte. Arrow indicates the direction of forward scan.

The anodic region of the CV of **2** exhibits four irreversible oxidation processes at onset potentials of 0.24, 0.65, 1.09 and 1.36 V due to the formation of the mixed-valence species $TTF^0/TTF^{\bullet+}$, $(TTF^{\bullet+})_2$, $TTF^{\bullet+}/TTF^{2+}$ and $(TTF^{2+})_2$, respectively (**Supplementary Figure 9**). Upon cycling, these features similarly diminish in current, likely due to degradation of the framework or irreversible structural rearrangements.

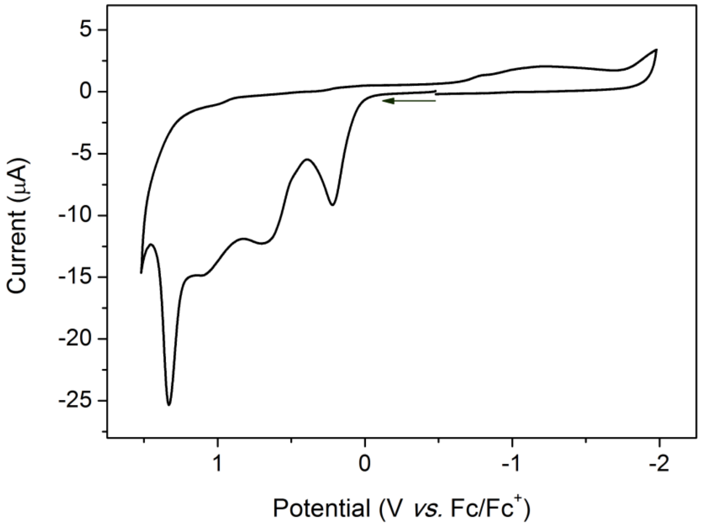

Supplementary Figure 9. Solid-state CV for **2** obtained at 100 mVs^{-1} in 0.1 M $TBAPF_6$ in CH_3CN supporting electrolyte. Arrow indicates the direction of forward scan.

4.2 Spectroelectrochemistry properties for **1**.

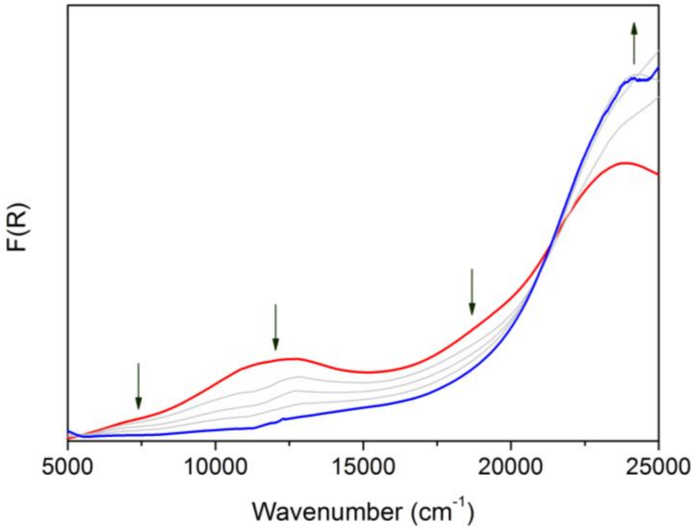

Supplementary Figure 10. Solid state Vis-NIR spectroelectrochemistry for **1** showing the oxidized compound at 1.30 V (red) and reduction back to its original state at 0 V (blue). Grey spectra correspond to the spectral transition over the applied potential range of 1.30-0 V. Experiment performed in 0.1 M $TBAPF_6$ in CH_3CN supporting electrolyte. Arrows indicate spectral change.

Reduction of the oxidized compound at 0 V saw a decrease in intensity at 7000, 12000 and 19000 cm^{-1} signalling the reversibility of the oxidation process. Interestingly, the band at 24000 cm^{-1} increases in intensity, possibly as a result of the stability of $TTF^{\bullet+}$ in the framework structure (**Supplementary Figures 10,11**).

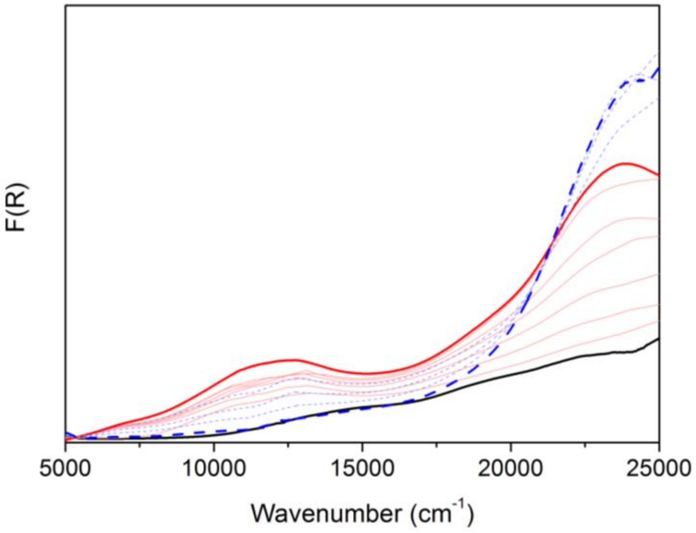

Supplementary Figure 11. An overlay of solid-state Vis-NIR spectroelectrochemical data for **1** of oxidation (applied potential 1.00-1.30 V; red solid) and reduction (potential returned to 0 V; blue

broken) processes. The spectrum for **1** under no bias is shown in black. Experiment performed in 0.1 M $TBAPF_6$ in CH_3CN supporting electrolyte.

4.3 Magnetic properties for **2**.

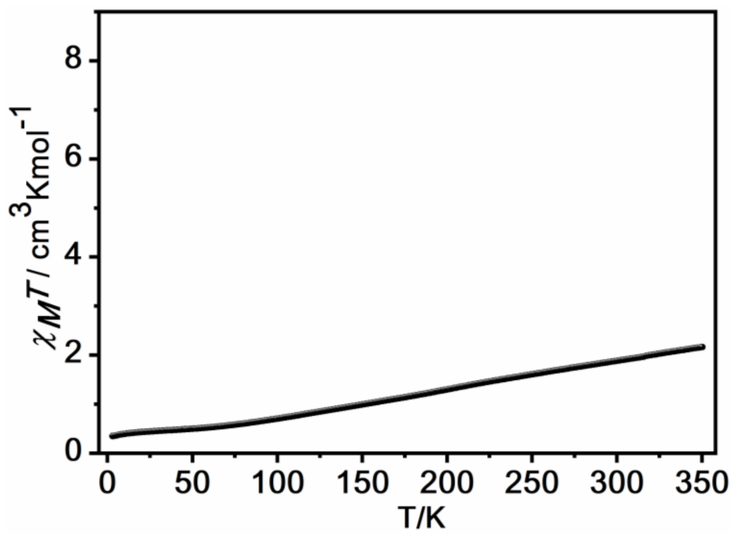

Supplementary Figure 12. Temperature dependence of the $\chi_M T$ in 1 kOe for **2**.

4.4 Fluorescence properties for TPPE, **1** and **2**.

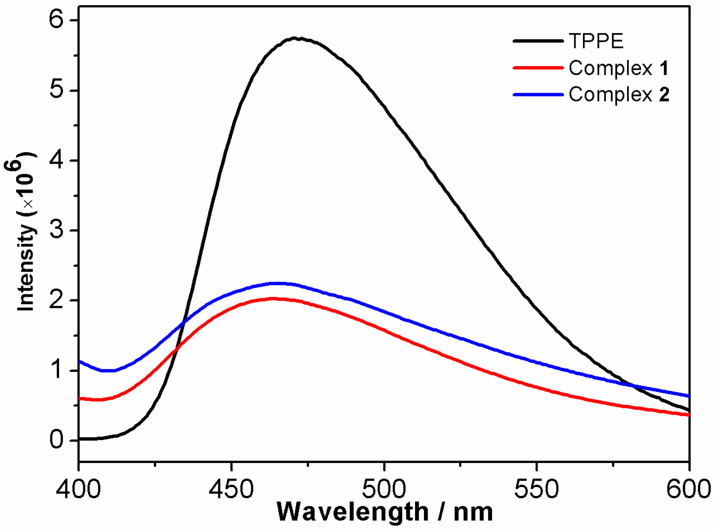

Supplementary Figure 13. Fluorescence emission spectra at room temperature for TPPE, **1** and **2** excited at 360 nm.

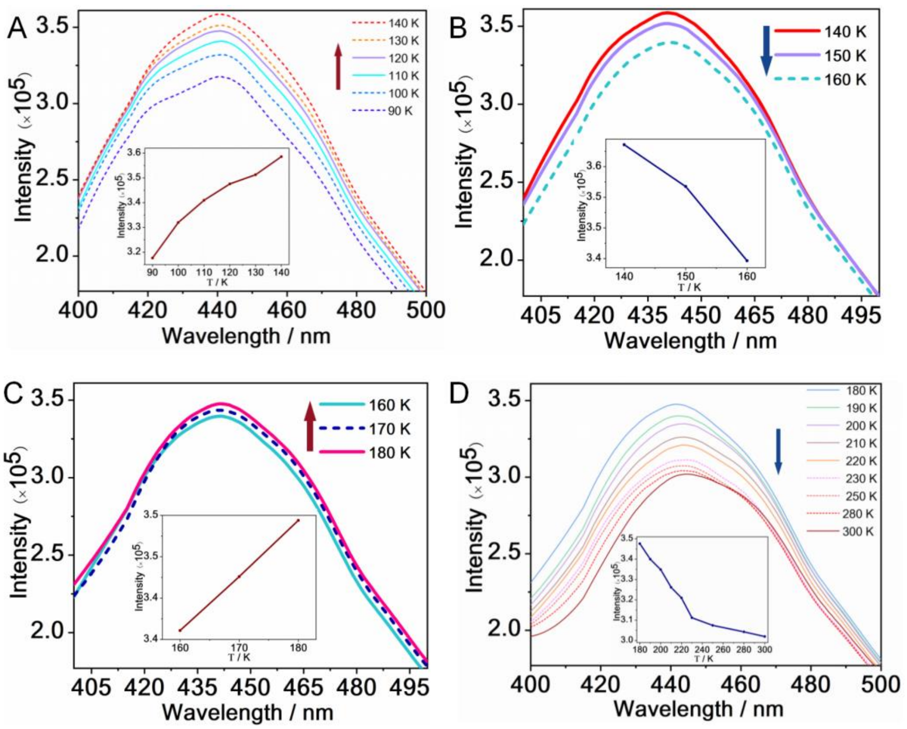

Supplementary Figure 14. Fluorescence spectra for **1** over the temperature range 90-140 K, 140-160 K, 160-180 K and 180-300 K (Inset: fluorescence intensity *vs* *T* plot) (A-D), respectively.

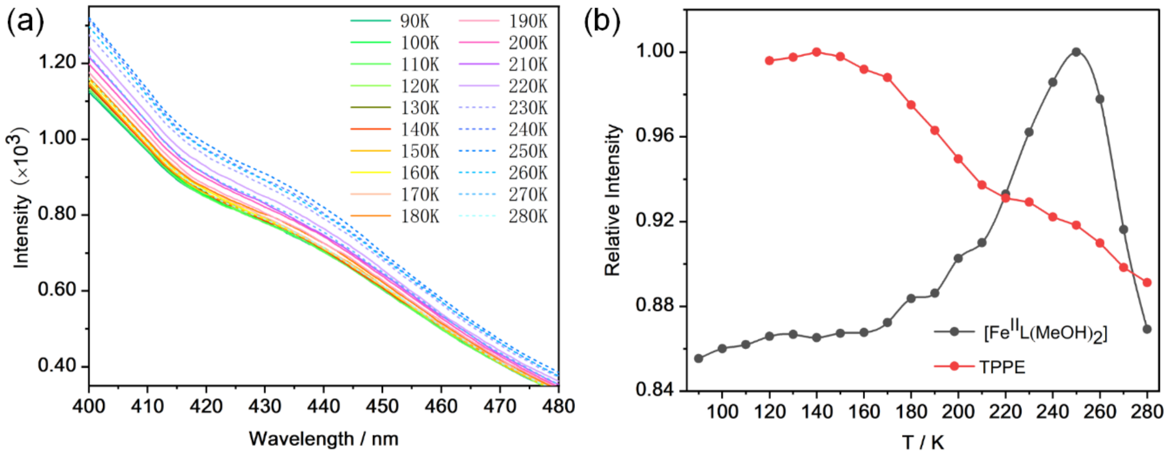

Supplementary Figure 15. The fluorescence spectra (a) and the fluorescence intensity *vs* T plot over the temperature range 90-280 K for **[Fe^{II}L(MeOH)$_2$]**, and **TPPE** (b).

4.5 The ^{1}H NMR spectrum for **H$_2$L**.

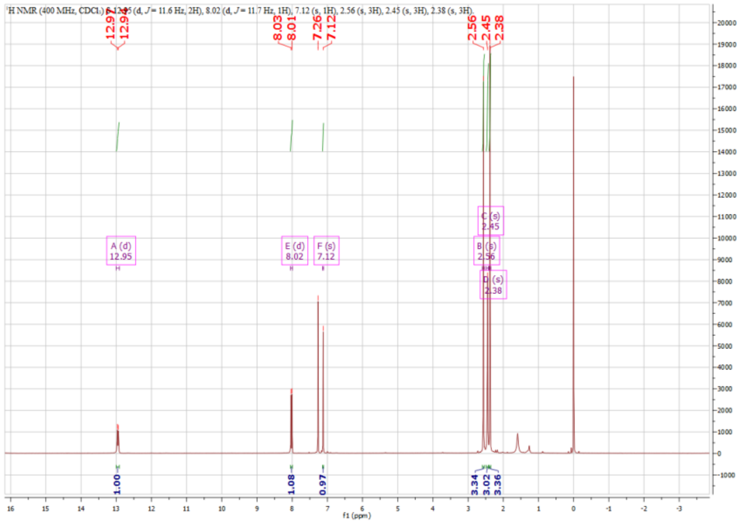

Supplementary Figure 16. The ^{1}H NMR spectrum for **H_2L** in $CDCl_3$.

5. References

1. (a) Gao, F., Cui, L., Liu, W., Hu, L., Zhong, Y.-W., Li, Y.-Z. and Zuo, J.-L. (2013). Seven-coordinate lanthanide sandwich-type complexes with a tetrathiafulvalene-fused schiff base ligand. *Inorg. Chem.* 52, 11164-11172; (b) Peng, Y.-H., Meng, Y.-F., Hu, L., Li, Q.-X., Li, Y.-Z., Zuo, J.-L. and You, X.-Z. (2010). Syntheses, structures, and magnetic properties of heterobimetallic clusters with tricyanometalate and π-conjugated ligands containing 1,3-dithiol-2-ylidene. *Inorg. Chem.* 49, 1905-1912.

2. Lochenie, C., Schötz, K., Panzer, F., Kurz, H., Maier, B., Puchtler, F., Agarwal, S., Köhler, A. and Weber, B. (2018). Spin-crossover iron(II) coordination polymer with fluorescent properties: correlation between emission properties and spin state. *J. Am. Chem. Soc.* 140, 700-709.

3. SAINT-Plus, version 6.02; Bruker analytical X-ray system, Madison, WI, 1999.

4. Sheldrick, G. M. (1996). SADABS, an empirical absorption correction program; Bruker analytical X-ray systems, Madison, WI.

5. Sheldrick, G. M. (2015). Crystal structure refinement with SHELXL. *Acta Crystallogr., Sect. C: Struct. Chem.* 71, 3-8.
