# Supplementary material for: Iron(II) Spin Crossover Coordination Polymers Derived From a Redox Active Equatorial Tetrathiafulvalene Schiff-Base Ligand
Source: Front Chem. 2021 Aug 2;9:692939. doi: 10.3389/fchem.2021.692939 (PMC8365465; doi:10.3389/fchem.2021.692939)

checkCIF/PLATON report

You have not supplied any structure factors. As a result the full set of tests cannot be run.

THIS REPORT IS FOR GUIDANCE ONLY. IF USED AS PART OF A REVIEW PROCEDURE FOR PUBLICATION, IT SHOULD NOT REPLACE THE EXPERTISE OF AN EXPERIENCED CRYSTALLOGRAPHIC REFEREE.

No syntax errors found. CIF dictionary Interpreting this report

Datablock: 1

```
Bond precision:  C-C = 0.0090 A          Wavelength=0.71073

Cell:           a=34.962(4)    b=25.670(3)        c=18.2005(19)
                alpha=90       beta=120.501(2)    gamma=90
Temperature:    123 K
```

	Calculated	Reported
Volume	14074(3)	14074(3)
Space group	C 2/c	C 2/c
Hall group	-C 2yc	-C 2yc
Moiety formula	C47 H38 Fe N4 O4 S6 [+ solvent]	?
Sum formula	C47 H38 Fe N4 O4 S6 [+ solvent]	C47 H38 Fe N4 O4 S6
Mr	971.02	971.02
Dx,g cm-3	0.917	0.917
Z	8	8
Mu (mm-1)	0.424	0.424
F000	4016.0	4016.0
F000’	4025.96	
h,k,lmax	41,30,21	41,30,21
Nref	12406	12389
Tmin,Tmax	0.873,0.923	0.892,0.911
Tmin’	0.873	

```
Correction method= # Reported T Limits: Tmin=0.892 Tmax=0.911
AbsCorr = MULTI-SCAN

Data completeness= 0.999          Theta(max)= 25.009

R(reflections)= 0.0871( 7313)     wR2(reflections)= 0.1991( 12389)

S = 1.169                   Npar= 566
```

The following ALERTS were generated. Each ALERT has the format
test-name_ALERT_alert-type_alert-level.
Click on the hyperlinks for more details of the test.

Alert level C

```
PLAT220_ALERT_2_C NonSolvent   Resd 1  C   Ueq(max)/Ueq(min) Range        3.2 Ratio
PLAT241_ALERT_2_C High   'MainMol' Ueq as Compared to Neighbors of         S1 Check
PLAT241_ALERT_2_C High   'MainMol' Ueq as Compared to Neighbors of         S2 Check
PLAT242_ALERT_2_C Low    'MainMol' Ueq as Compared to Neighbors of        C15 Check
PLAT242_ALERT_2_C Low    'MainMol' Ueq as Compared to Neighbors of        C17 Check
PLAT341_ALERT_3_C Low Bond Precision on  C-C Bonds ...............      0.009 Ang.
```

Alert level G

```
PLAT003_ALERT_2_G Number of Uiso or Uij Restrained non-H Atoms ...          1 Report
PLAT004_ALERT_5_G Polymeric Structure Found with Maximum Dimension          2 Info
PLAT012_ALERT_1_G N.O.K.   _shelx_res_checksum Found in CIF ......     Please Check
PLAT083_ALERT_2_G SHELXL Second Parameter in WGHT  Unusually Large      95.00 Why ?
PLAT128_ALERT_4_G Alternate Setting for Input Space Group     C2/c       I2/a Note
PLAT186_ALERT_4_G The CIF-Embedded .res File Contains ISOR Records          1 Report
PLAT606_ALERT_4_G Solvent Accessible VOID(S) in Structure ........          ! Info
PLAT860_ALERT_3_G Number of Least-Squares Restraints .............          6 Note
PLAT869_ALERT_4_G ALERTS Related to the Use of SQUEEZE Suppressed           ! Info
PLAT883_ALERT_1_G No Info/Value for _atom_sites_solution_primary .     Please Do !
PLAT933_ALERT_2_G Number of OMIT Records in Embedded .res File ...          4 Note
PLAT941_ALERT_3_G Average HKL Measurement Multiplicity ...........        3.2 Low
PLAT965_ALERT_2_G The SHELXL WEIGHT Optimisation has not Converged     Please Check
```

---

0 **ALERT level A** = Most likely a serious problem - resolve or explain
0 **ALERT level B** = A potentially serious problem, consider carefully
6 **ALERT level C** = Check. Ensure it is not caused by an omission or oversight
13 **ALERT level G** = General information/check it is not something unexpected

2 ALERT type 1 CIF construction/syntax error, inconsistent or missing data
9 ALERT type 2 Indicator that the structure model may be wrong or deficient
3 ALERT type 3 Indicator that the structure quality may be low
4 ALERT type 4 Improvement, methodology, query or suggestion
1 ALERT type 5 Informative message, check

Datablock: 2

Bond precision:	C-C = 0.0103 A		Wavelength=0.71073
Cell:	a=34.040(5)	b=27.578(3)	c=43.476(5)
	alpha=90	beta=112.554(3)	gamma=90
Temperature:	123 K		

	Calculated	Reported
Volume	37692(8)	37692(8)
Space group	C 2/c	C 2/c
Hall group	-C 2yc	-C 2yc
Moiety formula	C94 H76 Fe2 N8 O8 S12, C47 H37 Fe N4 O4 S6 [+ solvent]	?
Sum formula	C141 H113 Fe3 N12 O12 S18 [+ solvent]	C141 H113 Fe3 N12 O12 S18
Mr	2912.07	2912.06
Dx,g cm-3	1.026	1.026
Z	8	8
Mu (mm-1)	0.475	0.475
F000	12040.0	12040.0
F000'	12069.87	
h,k,lmax	40,32,51	40,32,51
Nref	33228	33131
Tmin,Tmax	0.867,0.901	0.880,0.901
Tmin'	0.847	

Correction method= # Reported T Limits: Tmin=0.880 Tmax=0.901
AbsCorr = MULTI-SCAN

Data completeness= 0.997 Theta(max)= 25.010

R(reflections)= 0.0929(18227) wR2(reflections)= 0.2165(33131)

S = 1.074 Npar= 1693

The following ALERTS were generated. Each ALERT has the format
test-name_ALERT_alert-type_alert-level.
Click on the hyperlinks for more details of the test.

Alert level C

```
PLAT220_ALERT_2_C NonSolvent   Resd 1  C   Ueq(max)/Ueq(min) Range        5.4 Ratio
PLAT220_ALERT_2_C NonSolvent   Resd 1  O   Ueq(max)/Ueq(min) Range        3.3 Ratio
PLAT221_ALERT_2_C Solv./Anion  Resd 2  C   Ueq(max)/Ueq(min) Range        5.2 Ratio
PLAT221_ALERT_2_C Solv./Anion  Resd 2  O   Ueq(max)/Ueq(min) Range        4.6 Ratio
PLAT222_ALERT_3_C NonSolvent Resd 1  H   Uiso(max)/Uiso(min) Range        6.0 Ratio
PLAT223_ALERT_4_C Solv./Anion  Resd 2  H   Ueq(max)/Ueq(min) Range        5.7 Ratio
PLAT242_ALERT_2_C Low    'MainMol' Ueq as Compared to Neighbors of         S8 Check
PLAT242_ALERT_2_C Low    'MainMol' Ueq as Compared to Neighbors of        S14 Check
PLAT242_ALERT_2_C Low    'MainMol' Ueq as Compared to Neighbors of        C65 Check
PLAT242_ALERT_2_C Low    'MainMol' Ueq as Compared to Neighbors of       C135 Check
PLAT242_ALERT_2_C Low    'MainMol' Ueq as Compared to Neighbors of         S1 Check
PLAT242_ALERT_2_C Low    'MainMol' Ueq as Compared to Neighbors of        C20 Check
PLAT242_ALERT_2_C Low    'MainMol' Ueq as Compared to Neighbors of        C23 Check
PLAT334_ALERT_2_C Small Aver. Benzene C-C Dist C86      -C88          1.37 Ang.
PLAT341_ALERT_3_C Low Bond Precision on  C-C Bonds ...............    0.01034 Ang.
PLAT410_ALERT_2_C Short Intra H...H Contact  H19      ..H24B     .     1.96 Ang.
                                                        x,y,z  =      1_555 Check
```

Alert level G

```
PLAT004_ALERT_5_G Polymeric Structure Found with Maximum Dimension          3 Info
PLAT012_ALERT_1_G N.O.K.   _shelx_res_checksum Found in CIF ......     Please Check
PLAT083_ALERT_2_G SHELXL Second Parameter in WGHT  Unusually Large     400.00 Why ?
PLAT606_ALERT_4_G Solvent Accessible VOID(S) in Structure ........          ! Info
PLAT720_ALERT_4_G Number of Unusual/Non-Standard Labels ..........         18 Note
PLAT794_ALERT_5_G Tentative Bond Valency for Fe3       (III)     .       3.97 Info
PLAT869_ALERT_4_G ALERTS Related to the Use of SQUEEZE Suppressed           ! Info
PLAT883_ALERT_1_G No Info/Value for _atom_sites_solution_primary .     Please Do !
PLAT933_ALERT_2_G Number of OMIT Records in Embedded .res File ...          3 Note
PLAT941_ALERT_3_G Average HKL Measurement Multiplicity ...........        2.4 Low
PLAT965_ALERT_2_G The SHELXL WEIGHT Optimisation has not Converged     Please Check
```

```
   0 ALERT level A = Most likely a serious problem - resolve or explain
   0 ALERT level B = A potentially serious problem, consider carefully
  16 ALERT level C = Check. Ensure it is not caused by an omission or oversight
  11 ALERT level G = General information/check it is not something unexpected

   2 ALERT type 1 CIF construction/syntax error, inconsistent or missing data
  16 ALERT type 2 Indicator that the structure model may be wrong or deficient
   3 ALERT type 3 Indicator that the structure quality may be low
   4 ALERT type 4 Improvement, methodology, query or suggestion
   2 ALERT type 5 Informative message, check
```

Datablock: H2L

```
Bond precision:  C-C = 0.0071 A          Wavelength=0.71073

Cell:            a=5.0923(5)     b=15.4249(17)      c=38.309(4)
                 alpha=90        beta=91.071(3)     gamma=90
Temperature:     296 K
```

	Calculated	Reported
Volume	3008.6(5)	3008.6(5)
Space group	P 21/n	P2(1)/n
Hall group	-P 2yn	?
Moiety formula	C24 H24 N2 O4 S6, C2 H3 N	?
Sum formula	C26 H27 N3 O4 S6	C26 H27 N3 O4 S6
Mr	637.87	637.87
Dx,g cm-3	1.408	1.408
Z	4	4
Mu (mm-1)	0.491	0.491
F000	1328.0	1328.0
F000’	1331.41	
h,k,lmax	6,20,50	6,20,49
Nref	7068	7005
Tmin,Tmax	0.889,0.929	0.889,0.929
Tmin’	0.876	

```
Correction method= # Reported T Limits: Tmin=0.889 Tmax=0.929
AbsCorr = MULTI-SCAN

Data completeness= 0.991          Theta(max)= 27.700

R(reflections)= 0.0882( 4208)     wR2(reflections)= 0.1872( 7005)

S = 1.096                   Npar= 367
```

The following ALERTS were generated. Each ALERT has the format
test-name_ALERT_alert-type_alert-level.
Click on the hyperlinks for more details of the test.

Alert level C

```
PLAT220_ALERT_2_C NonSolvent   Resd 1  C   Ueq(max)/Ueq(min) Range        3.4 Ratio
PLAT222_ALERT_3_C NonSolvent Resd 1  H   Uiso(max)/Uiso(min) Range        4.7 Ratio
PLAT242_ALERT_2_C Low    'MainMol' Ueq as Compared to Neighbors of         S5 Check
PLAT242_ALERT_2_C Low    'MainMol' Ueq as Compared to Neighbors of         C4 Check
PLAT242_ALERT_2_C Low    'MainMol' Ueq as Compared to Neighbors of        C15 Check
PLAT244_ALERT_4_C Low    'Solvent' Ueq as Compared to Neighbors of        C26 Check
PLAT250_ALERT_2_C Large U3/U1 Ratio for Average U(i,j) Tensor ....        2.1 Note
PLAT260_ALERT_2_C Large Average Ueq of Residue Including        N1      0.106 Check
PLAT340_ALERT_3_C Low Bond Precision on  C-C Bonds ...............    0.00711 Ang.
```

Alert level G

```
PLAT005_ALERT_5_G No Embedded Refinement Details Found  in the CIF     Please Do !
PLAT066_ALERT_1_G Predicted and Reported Tmin&Tmax Range Identical        ? Check
PLAT899_ALERT_4_G SHELXL97  is Deprecated and Succeeded by SHELXL/      2018 Note
```

---

0 **ALERT level A** = Most likely a serious problem - resolve or explain
0 **ALERT level B** = A potentially serious problem, consider carefully
9 **ALERT level C** = Check. Ensure it is not caused by an omission or oversight
3 **ALERT level G** = General information/check it is not something unexpected

1 ALERT type 1 CIF construction/syntax error, inconsistent or missing data
6 ALERT type 2 Indicator that the structure model may be wrong or deficient
2 ALERT type 3 Indicator that the structure quality may be low
2 ALERT type 4 Improvement, methodology, query or suggestion
1 ALERT type 5 Informative message, check

Datablock: FeLCH3OH2

```
Bond precision:   C-C = 0.0074 A          Wavelength=0.71073

Cell:             a=15.835(5)      b=28.144(9)          c=8.961(3)
                  alpha=90         beta=100.929(6)      gamma=90
Temperature:      296 K
```

	Calculated	Reported
Volume	3921(2)	3921(2)
Space group	P 21/c	P 21/c
Hall group	-P 2ybc	-P 2ybc
Moiety formula	C26 H30 Fe N2 O6 S6, 4(C H4 O)	?
Sum formula	C30 H46 Fe N2 O10 S6	C30 H46 Fe N2 O10 S6
Mr	842.90	842.90
Dx,g cm-3	1.428	1.428
Z	4	4
Mu (mm-1)	0.757	0.757
F000	1768.0	1768.0
F000’	1773.04	
h,k,lmax	19,35,11	19,35,11
Nref	8110	7984
Tmin,Tmax	0.860,0.893	0.860,0.893
Tmin’	0.860	

Correction method= # Reported T Limits: Tmin=0.860 Tmax=0.893
AbsCorr = MULTI-SCAN

Data completeness= 0.984 Theta(max)= 26.484

R(reflections)= 0.0749(5559) wR2(reflections)= 0.2101(7984)

S = 1.038 Npar= 454

The following ALERTS were generated. Each ALERT has the format
test-name_ALERT_alert-type_alert-level.
Click on the hyperlinks for more details of the test.

Alert level C

```
PLAT220_ALERT_2_C NonSolvent   Resd 1  C   Ueq(max)/Ueq(min) Range        3.6 Ratio
PLAT222_ALERT_3_C NonSolvent Resd 1  H   Uiso(max)/Uiso(min) Range        4.1 Ratio
PLAT260_ALERT_2_C Large Average Ueq of Residue Including        O7      0.158 Check
PLAT260_ALERT_2_C Large Average Ueq of Residue Including        O8      0.175 Check
PLAT260_ALERT_2_C Large Average Ueq of Residue Including        O9      0.171 Check
PLAT260_ALERT_2_C Large Average Ueq of Residue Including       O10      0.238 Check
PLAT341_ALERT_3_C Low Bond Precision on  C-C Bonds ...............    0.00739 Ang.
PLAT410_ALERT_2_C Short Intra H...H Contact  H6A      ..H8A      .       1.96 Ang.
                                                      x,y,z  =      1_555 Check
PLAT480_ALERT_4_C Long H...A H-Bond Reported H25A     ..S2       .       2.91 Ang.
PLAT480_ALERT_4_C Long H...A H-Bond Reported H24A     ..O1       .       2.65 Ang.
PLAT480_ALERT_4_C Long H...A H-Bond Reported H22B     ..S5       .       2.92 Ang.
PLAT480_ALERT_4_C Long H...A H-Bond Reported H1C      ..S5       .       3.00 Ang.
```

Alert level G

```
PLAT002_ALERT_2_G Number of Distance or Angle Restraints on AtSite         11 Note
PLAT003_ALERT_2_G Number of Uiso or Uij Restrained non-H Atoms ...          8 Report
PLAT007_ALERT_5_G Number of Unrefined Donor-H Atoms ..............          6 Report
PLAT066_ALERT_1_G Predicted and Reported Tmin&Tmax Range Identical          ? Check
```

```
PLAT083_ALERT_2_G SHELXL Second Parameter in WGHT  Unusually Large      17.37 Why ?
PLAT172_ALERT_4_G The CIF-Embedded .res File Contains DFIX Records          3 Report
PLAT178_ALERT_4_G The CIF-Embedded .res File Contains SIMU Records          1 Report
PLAT794_ALERT_5_G Tentative Bond Valency for Fe1       (II)      .       2.36 Info
PLAT860_ALERT_3_G Number of Least-Squares Restraints .............         91 Note
PLAT883_ALERT_1_G No Info/Value for _atom_sites_solution_primary .     Please Do !
PLAT933_ALERT_2_G Number of OMIT Records in Embedded .res File ...         76 Note
PLAT941_ALERT_3_G Average HKL Measurement Multiplicity ...........        3.0 Low
PLAT965_ALERT_2_G The SHELXL WEIGHT Optimisation has not Converged     Please Check
```

```
   0 ALERT level A = Most likely a serious problem - resolve or explain
   0 ALERT level B = A potentially serious problem, consider carefully
  12 ALERT level C = Check. Ensure it is not caused by an omission or oversight
  13 ALERT level G = General information/check it is not something unexpected

   2 ALERT type 1 CIF construction/syntax error, inconsistent or missing data
  11 ALERT type 2 Indicator that the structure model may be wrong or deficient
   4 ALERT type 3 Indicator that the structure quality may be low
   6 ALERT type 4 Improvement, methodology, query or suggestion
   2 ALERT type 5 Informative message, check
```

It is advisable to attempt to resolve as many as possible of the alerts in all categories. Often the minor alerts point to easily fixed oversights, errors and omissions in your CIF or refinement strategy, so attention to these fine details can be worthwhile. In order to resolve some of the more serious problems it may be necessary to carry out additional measurements or structure refinements. However, the purpose of your study may justify the reported deviations and the more serious of these should normally be commented upon in the discussion or experimental section of a paper or in the "special_details" fields of the CIF. checkCIF was carefully designed to identify outliers and unusual parameters, but every test has its limitations and alerts that are not important in a particular case may appear. Conversely, the absence of alerts does not guarantee there are no aspects of the results needing attention. It is up to the individual to critically assess their own results and, if necessary, seek expert advice.

Publication of your CIF in IUCr journals

A basic structural check has been run on your CIF. These basic checks will be run on all CIFs submitted for publication in IUCr journals (*Acta Crystallographica*, *Journal of Applied Crystallography*, *Journal of Synchrotron Radiation*); however, if you intend to submit to *Acta Crystallographica Section C* or *E* or *IUCrData*, you should make sure that full publication checks are run on the final version of your CIF prior to submission.

Publication of your CIF in other journals

Please refer to the *Notes for Authors* of the relevant journal for any special instructions relating to CIF submission.

PLATON version of 10/08/2020; check.def file version of 06/08/2020

Datablock 1 - ellipsoid plot

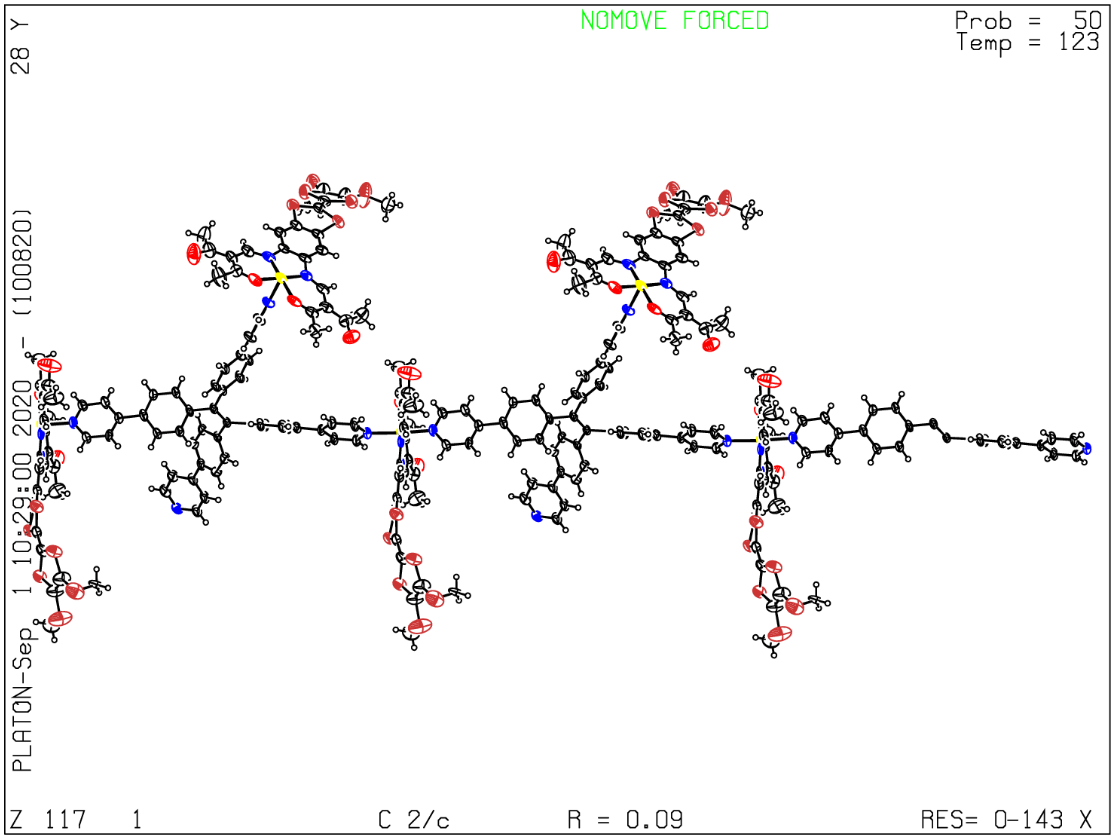

Datablock 2 - ellipsoid plot

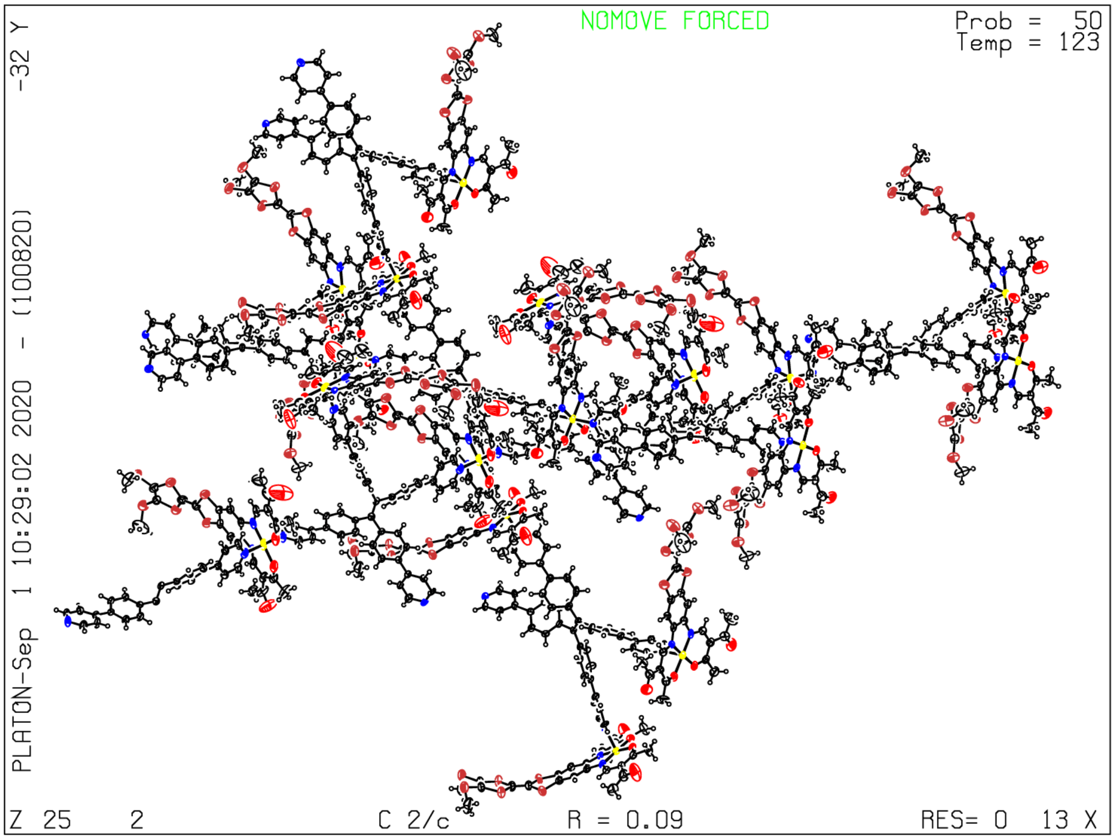

Datablock H2L - ellipsoid plot

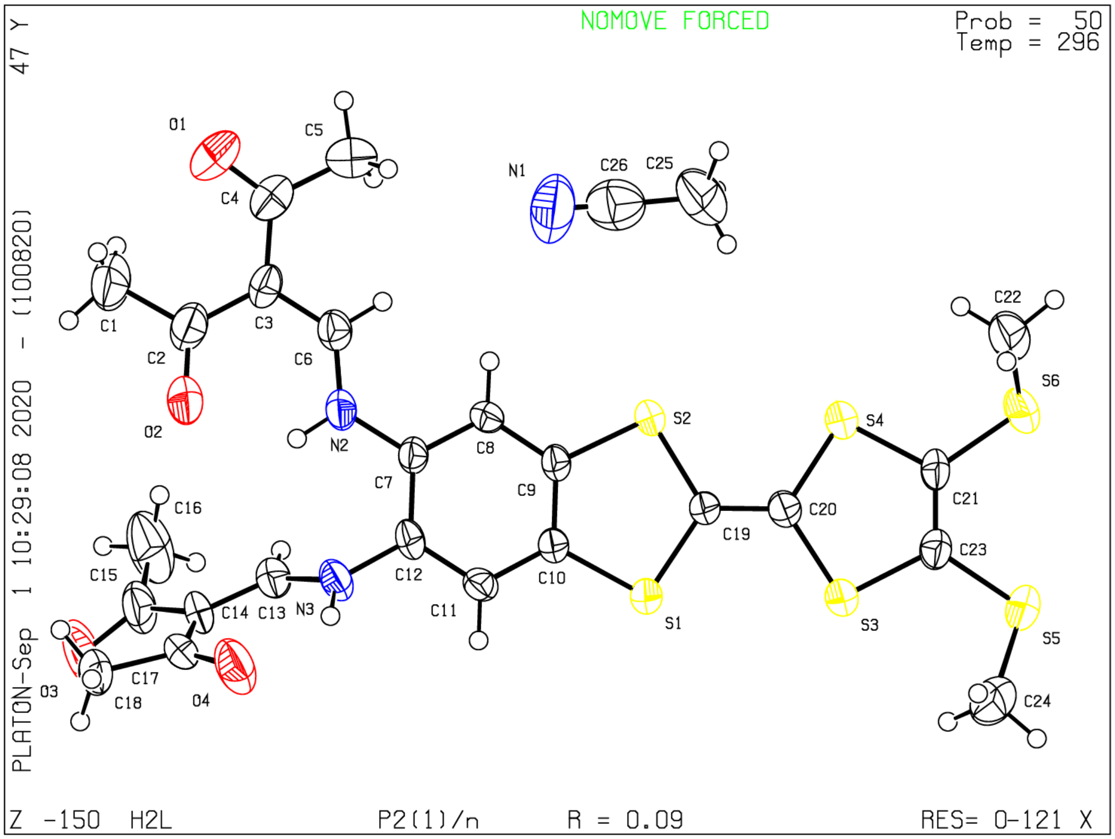

Datablock FeLCH3OH2 - ellipsoid plot

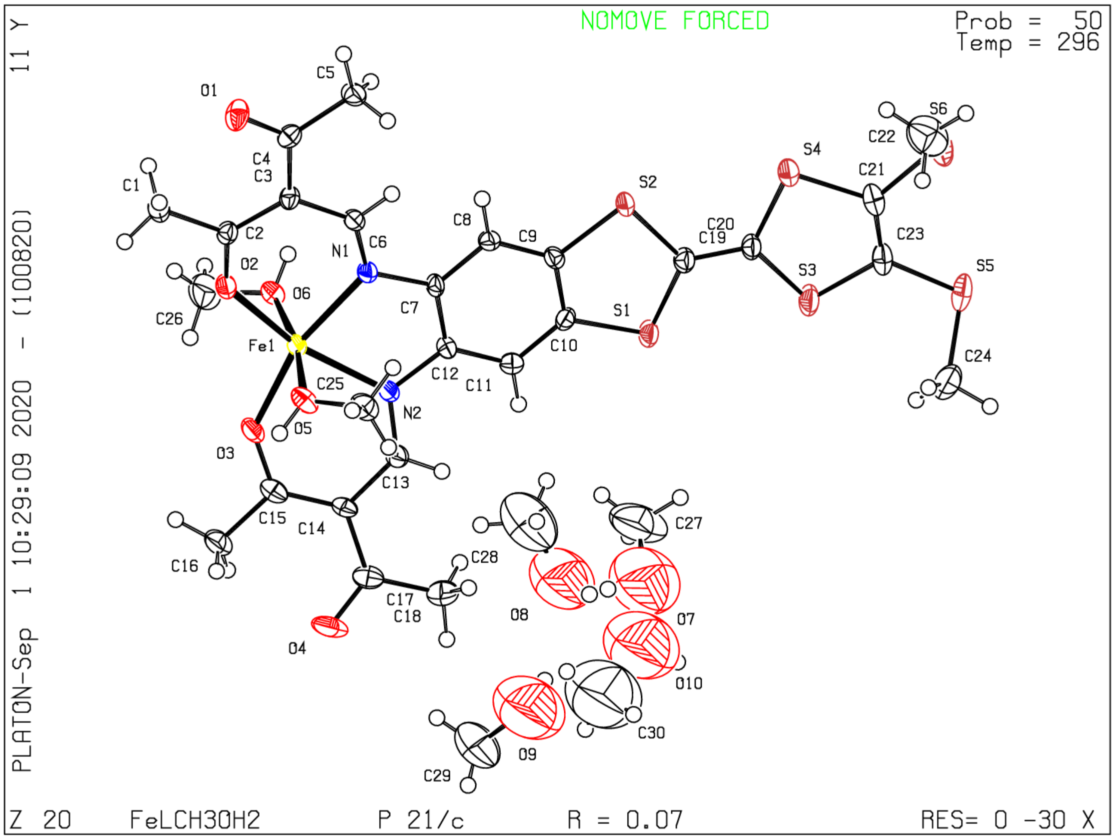

Supplement: Supplementary file 2 [file DataSheet1.PDF]
